# Effect of PHF-1 hyperphosphorylation on the seeding activity of C-terminal Tau fragments

**DOI:** 10.1038/s41598-025-91867-3

**Published:** 2025-03-22

**Authors:** Léa El Hajjar, Emmanuelle Boll, François-Xavier Cantrelle, Clarisse Bridot, Isabelle Landrieu, Caroline Smet-Nocca

**Affiliations:** 1 Inserm, CHU Lille, Institut Pasteur de Lille, U1167 - RID-AGE - Risk Factors and Molecular Determinants of Aging-Related Diseases, Univ. Lille, Lille, 59000 France; 2 CNRS EMR9002 Integrative Structural Biology, Lille, 59000 France; 3 Inserm U1167/Institut Pasteur de Lille, 1 rue Professeur Calmette, BP245, Lille, 59019 France

**Keywords:** Microtubule-associated tau protein, Tau fragments, Seeds, Fibrillar aggregation, Phosphorylation, GSK3*β*, NMR spectroscopy, Solution-state NMR, Alzheimer's disease

## Abstract

Tau proteins as neurofibrillary tangles are one of the molecular hallmarks of Alzheimer’s disease (AD) and play a central role in tauopathies, a group of age-related neurodegenerative disorders. The filament cores from diverse tauopathies share a common region of tau consisting of the R3-R4 microtubule-binding repeats and part of the C-terminal domain, but present a structural polymorphism. Unlike the fibril structure, the PTM signature of tau found in neuronal inclusions, more particularly hyperphosphorylation, is variable between individuals with the same tauopathy, giving rise to diverse strains with different seeding properties that could modulate the aggressiveness of tau pathology. Here, we investigate the conformation, function and seeding activity of two tau fragments and their GSK3*β-*phosphorylated variants. The R2Ct and R3Ct fragments encompass the aggregation-prone region of tau starting at the R2 and R3 repeats, respectively, and the full C-terminal domain including the PHF-1 epitope (S396, S400, S404), which undergoes a triple phosphorylation upon GSK3*β* activity. We found that the R3Ct fragment shows both a greater loss of function and pathological activity in seeding of aggregation than the R2Ct fragment which imposes a cross-seeding barrier. PHF-1 hyperphosphorylation induces a local conformational change with a propensity to adopt a β-sheet conformation in the region spanning residues 392–402, and exacerbates the seeding ability of fragments to induce aggregation by overcoming a cross-seeding barrier between tau variants.

**Supplementary Information:**

The online version contains supplementary material available at 10.1038/s41598-025-91867-3.

## Introduction

**Fig. 1:**
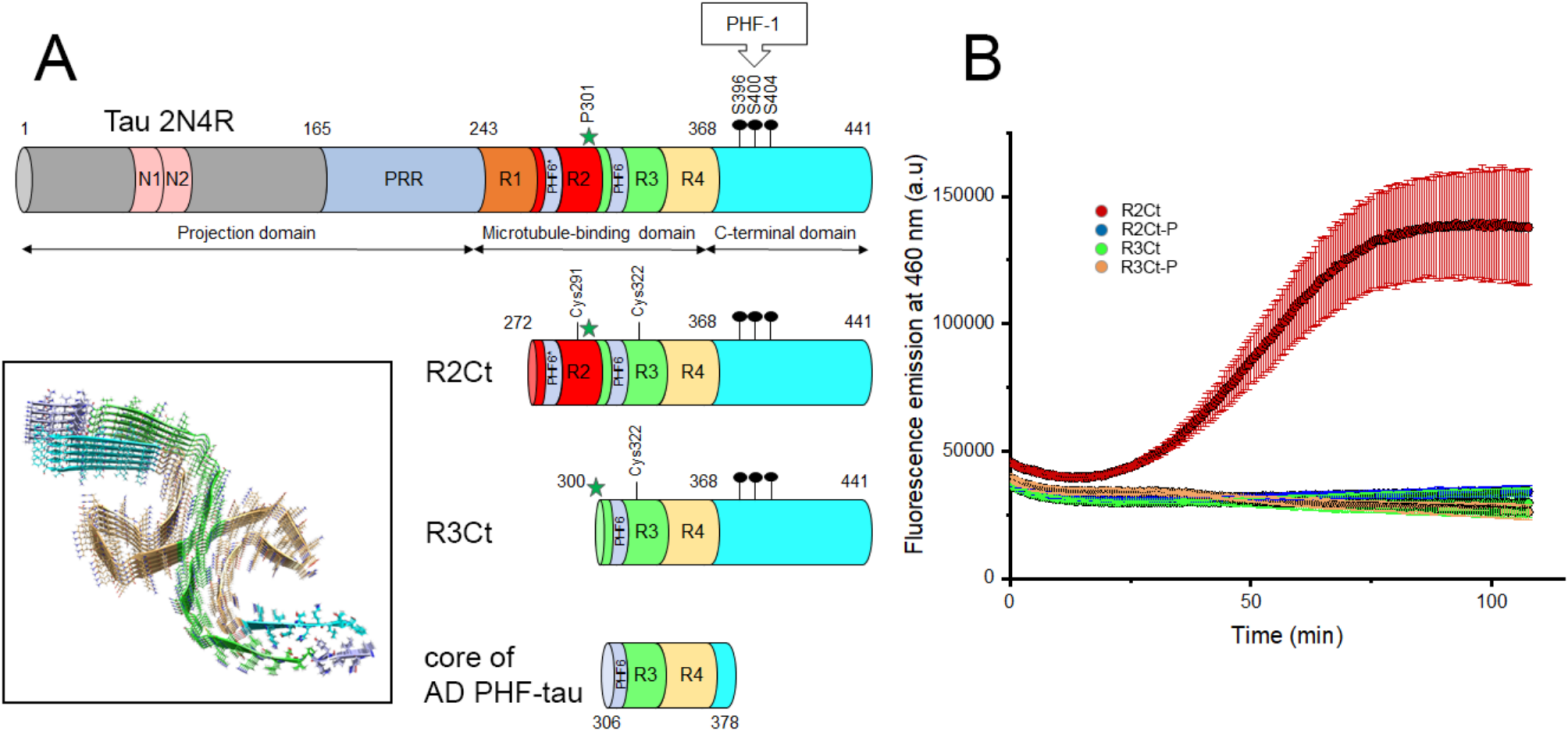
Schematic representation of domains of tau protein, R2Ct and R3Ct fragments used in this study, and activity on tubulin polymerization. (**A**) Sequences of tau 2N4R isoform, R2Ct and R3Ct fragments indicating the different domains encompassed in each protein. PHF6* (275-VQIINK-280) and PHF6 (306-VQIVYK-311) sequences in R2 and R3 repeats, respectively, correspond to hexapeptide motifs that have been described as nuclei of tau fibrillar aggregation. PHF-1 indicated by black dots is a phospho-epitope of the C-terminal domain (S396/S400/S404) found in tau inclusions from AD brains which is generated by the kinase activity either of GSK3β alone or combination of CDK2/cyclin A and GSK3β. The P301 site indicated by a green star is a missense mutation site (P301L or P301S) causing fronto-temporal dementia with Parkinsonism linked to chromosome-17. The core of PHF-tau filaments from AD brains (inset, PDB ID: 5O3L) is shown^43^. (**B**) Activity of tau fragments in a tubulin polymerization assay as shown by kinetics of tubulin polymerization in the presence of tau fragments R2Ct (red), R3Ct (green), and their GSK3β phosphorylated variants R2Ct-P (blue) and R3Ct-P (orange). Data are plotted as mean ± standard error to the mean (SEM) of experimental replicates (*n* = 3).

Tauopathies are a group of neurodegenerative diseases characterized by the presence of pathological inclusions or aggregates of the microtubule-associated protein tau^1–3^. The human tau protein, encoded by the *MAPT* gene, is an intrinsically disordered protein (IDP) which plays a critical role in microtubule (MT) assembly and stabilization within neurons^4,5^. However, tau has also been shown to act as a scaffolding protein with many other functions not restricted to MT binding and regulation of cytoskeletal plasticity^6^. Tau protein consists of four regions - an N-terminal projection domain, a proline-rich domain (PRD), a microtubule-binding domain composed of 3 or 4 microtubule-binding repeats (MTBR), and a C-terminal domain (Fig. 1A). Six tau isoforms exist in the adult human brain as a result of alternative splicing, distinguished by the combination of three (3R) or four (4R) MTBRs with zero, one, or two N-terminal inserts (0 N, 1 N, 2 N, respectively), with an equal ratio of 3R and 4R isoforms. The MTBR consists of conserved repeat sequences of 18 residues, each separated by less conserved inter-repeat sequences of 13 or 14 residues (from R1 to R4), in which exon 10 encoding the second repeat (R2) is alternatively spliced^7^. Functional discrepancies have been associated with different tau isoforms, which are differentially distributed in neuronal subcellular compartments^8–10^. One of the most important functional differences lies in the higher affinity of 4R isoforms for MT binding which are also more efficient in promoting tubulin assembly.

Furthermore, the balance of 3R:4R isoforms is critical for tau function as some tauopathies are associated with a 3R:4R imbalance. The MTBR is not only critical for tau interaction with MTs, but also contributes to tau aggregation into filaments and neurofibrillary tangles (NFTs) observed in Alzheimer’s disease (AD) and other tauopathies^11–16^. The R2 and R3 domains each contain an hexapeptide sequence, PHF6* (275-VQIINK-280) and PHF6 (306-VQIVYK-311), respectively, which constitute two key fibril-forming regions, while cysteine residues C291 in R2 and C322 in R3 have been shown to accelerate nucleation in the fibrillization process (Fig. 1A)^17–24^. Recently, a mechanism of liquid-liquid phase separation (LLPS) has been proposed to promote the assembly of intracellular amyloid into liquid droplets gathering high concentrations of tau with cofactors such as RNA^25,26^. The formation of amyloid-like filaments originates from protein misfolding, but abnormal phosphorylation, or hyperphosphorylation, has also been shown to be a common feature of tau proteins found in inclusions of various tauopathies^27–29^. Several other reversible posttranslational modifications (PTMs) including glycosylation, acetylation, ubiquitination, methylation, glycation, and oxidation have also been associated with the regulation of tau pathophysiological functions. In addition, irreversible proteolysis catalyzed by numerous proteases generates a range of tau fragments with more or less toxicity^30,31^. Some of them have been highlighted for their ability to exacerbate tau aggregation and could be precursors in the mechanism of filament formation^32–34^. Specific tau PTMs and fragments have been found in patient cerebrospinal fluid and plasma, suggesting that these species may serve as disease biomarkers^31^. In addition, there is growing evidence that tau aggregation processes share similarities with prions, in which tau species with abnormal conformations can seed the aggregation of normal tau proteins through a prion-like mechanism triggering a self-amplifying cascade in different brain areas^35–38^. These pathological species, which can be transmitted between anatomically connected neurons, therefore contributing to the spread of tau pathology in a spatio-temporally defined manner, remain to be described at the molecular level. The precise mechanisms of tau propagation, involving cellular secretion/uptake or transcellular transfer of tau and seeding of fibrillation in recipient cells, are still to be defined as well^39^. In this context, reversible chemical PTMs and the production of proteolytic fragments may be critical steps in both the aggregation and cell-to-cell spreading of tau in neurodegeneration, and may be responsible for the diversity of tau strains associated with distinct tauopathies^40,41^.

Tauopathies differ in (i) the isoforms of tau (3R, 4R, or both) found embedded into amyloid fibrils, (ii) the presence or absence of mutations in the *MAPT* gene, particularly within the MTBR, such as the P301L or P301S mutations in frontotemporal dementia linked to Parkinsonism (FTDP), or in intronic regions affecting RNA splicing, (iii) the folds of the filament core, and (iv) the spatiotemporal patterns of tau pathology propagation^42^. Tau filament structures from different tauopathies revealed by cryo-EM at near-atomic resolution share a common ordered core region comprising R3, R4, and a short sequence of the C-terminal domain, but differ in the extent and composition of the region N-terminal to the R3 repeat. This heterogeneity, together with that of PTMs, cofactors and proteolytic fragments, that may be part of the fibrillar inclusions, is responsible for the structural polymorphism of tau fibrils. In AD, an equal mixture of 3R and 4R tau isoforms is found in both paired helical and straight tau filaments (PHFs and SFs, respectively), which are composed of two protofilaments sharing the same cross *β*-helix structure but differing in the protofilament assembly^43^. Notably, tau fibrils from Alzheimer’s patients share a common structure, suggesting a common mechanism of self-assembly. Conveniently, heparin and other polyanionic cofactors have been instrumental to mimic pathophysiological tau filaments in vitro, which has been useful in a range of studies investigating tau aggregation mechanisms, seeding in cellular models or other pathological functions, as well as finding a way to interfere with them, such as for the screening of tau aggregation inhibitors^44–49^. However, fibrils assembled with heparin, although they form structures that appear similar to PHFs in the form of twisted filaments in transmission electron microscopy (TEM), present heterogeneous structures, none of which share the same atomic fold as AD filaments^43,50,51^.

Interestingly, only 20% of the tau sequence (according to the longest 2N4R isoform) is embedded in the fibril core which comprises residues 306–378. The filament core is surrounded by a “fuzzy coat” formed by the disordered N- and C termini (Fig. 1A)^52^. Those regions flanking the organized amyloid core are known to regulate tau functions, interactions and aggregation properties by modulating binding to tubulin/MTs, the rate of fibrillar assembly, toxicity and LLPS properties^33,53,54^. The “fuzzy coat” remains accessible within the amyloid fibril and may still be targeted by posttranslational modifying and proteolytic enzymes^52,55–57^. Although hyperphosphorylation is closely associated with tau pathology in diverse tauopathies, the structural details of tau filaments from cryo-EM do not provide any information on the sites and mechanistic role of phosphorylation, which is mainly found within the N- and C-terminal regions flanking the amyloid core^29,58–61^. Thus, whether PTM diversity or site-specific modifications in the tau sequence play a critical role in the formation of amyloid fibrils, liquid droplets or pathological species that are transmitted between neurons remains a matter of debate. PTM heterogeneity could either be consistent with their irrelevance to the mechanism of tau fibrillation, while pathology would be predominantly driven by disease-specific amyloid folds that are conserved among patients. This key question could be partially addressed by chemical ligation approaches to produce homogenous PTMs in semisynthetic proteins amenable to in vitro fibrillation^47,62–66^.

In AD, tau PTM signatures, and particularly the hyperphosphorylated species of tau, have been found to be heterogenous and patient-specific, in contrast to the atomic structure of tau amyloid cores, which is faithfully reproduced between patients. Some PTM sites have been associated with enhanced seeding activity which correlates with disease aggressiveness^56^. Thus, distinct PTM signatures result in different strains with different seeding properties in the propagation of tau pathology in the brain, underscoring the importance of understanding the role of specific PTMs in tau pathophysiological functions. A recent study has highlighted a range of tau fragments capable of self-associating in vitro under specific conditions to form filaments morphologically similar to those observed in patients with AD and chronic traumatic encephalopathy (CTE), as shown by cryo-EM^53,67^. In contrast, the N- and C-terminal regions of tau have been shown to strongly inhibit, or completely prevent, filament assembly. A minimal tau fragment (297–391) has been identified that produces filaments structurally identical to AD fibrils and is able to recruit full-length tau to filaments. Oligomers derived from this fragment are internalized into cells and induce endogenous tau aggregation^53,68,69^. In addition, pseudo-phosphorylations at the C-terminal PHF-1 epitope (S396D, S400D, T403D, S404D) in the enlarged (297–408) fragment partially reproduce the AD fold, suggesting a role for PHF-1 phosphorylation in counteracting the inhibitory effect of the C-terminal domain^53^.

Characterization of the pathological tau species is essential to unravel the molecular processes leading to tau fibrillization. The GSK3*β* kinase has been shown to be closely associated with tau hyperphosphorylation in neurodegenerative diseases, pointing to a potential role for GSK3*β* activity in tau toxicity^70^. As we have previously shown, GSK3*β* is involved in hyperphosphorylation of the PHF-1 epitope (pS396/pS400/pS404, hereafter referred to as PHF1-3P; Figures S1 and S2) without the need for priming, and regulation of GSK3*β* kinase activity modulates PHF1-3P levels^71–73^. In our study, we evaluated the effect of *bona fide* homogeneous GSK3*β* phosphorylation of PHF-1 on the seeding activity of two tau fragments encompassing the entire C-terminal region starting from either the R2 or R3 repeat, namely tau R2Ct (272–441) and R3Ct (300–441), respectively (Fig. 1A). These fragments and their PHF-1 phosphorylated variants have not only lost their ability to polymerize tubulin, but also undergo a conformational change upon phosphorylation, and form small fibrillar aggregates that can seed tau aggregation. We have systematically examined the seeding and cross-seeding activity of the two fragments and their phosphorylated variants on monomeric R2Ct and R3Ct fragments, phosphorylated or not, and on 2N4R tau proteins. We have found that R3Ct forms short fibrillar seeds with higher seeding efficiency than R2Ct, and that PHF-1 hyperphosphorylation of both fragments increases their seeding activity on tau-P301L mutant protein.

## Results and discussion

### Loss-of-function of R2Ct and R3Ct fragments on tubulin polymerization

We have produced recombinant R2Ct and R3Ct tau fragments in *E. coli*, and their respective GSK3*β*-phosphorylated variants R2Ct-P and R3Ct-P, respectively. As controls, 2N4R tau proteins (tau-S262A and tau-PHF1 mutants^71^) were produced and phosphorylated with the same protocol. Each fragment was incubated at 20 *µ*M with 20 *µ*M of tubulin at 37 °C in polymerization buffer. The non-phosphorylated R2Ct fragment promotes polymerization, however in a lesser extent than full-length 2N4R tau (Fig. 1B, Figure S3A), and this ability is lost upon GSK3*β* phosphorylation. In contrast, the R3Ct fragment, phosphorylated or not, is not able to promote tubulin polymerization (Fig. 1B). Moreover, in the 2N4R tau isoform, similar phosphorylation restricted to the PHF-1 epitope in a triple phosphorylated state (in the tau-PHF1 mutant), here provided by a sequential phosphorylation of CDK2/cyclin A and GSK3*β*, only decreases kinetics of tubulin polymerization, but does not fully prevent it. In contrast, phosphorylation by CDK2/cyclin A alone, providing heterogeneously phosphorylated proteoforms on pS404 (90%) and pS396 (40%), does not change the tubulin polymerization rate related to the non-phosphorylated tau protein (Figure S3 B, D). In full-length tau (tau-S262A), GSK3*β* phosphorylation after CDK2 priming, leading to hyperphosphorylated forms on more than 14 sites, completely abrogate tubulin polymerization (Figure S3 C, E). Together our data indicate that a minimal number of MTBR, possibly together with the flanking proline-rich regulatory region, are required to counteract the loss of tubulin polymerization activity mediated by PHF-1 phosphorylation.

### Conformational changes of tau fragments induced by GSK3*β* phosphorylation of PHF-1 epitope

We next investigated the effect of phosphorylation on the conformation of R2Ct and R3Ct fragments by high-resolution NMR spectroscopy as conformational changes may have critical implications in interactions of tau with microtubules or other binding partners, in regulating tau function in tubulin polymerization or self-assembly into fibrils^74–76^. One of the major challenges in IDPs’ structural biology, which, unlike globular proteins, cannot be represented as a limited ensemble of conformers, is that IDPs are characterized by a large number of disordered states due to their high conformational heterogeneity. The representation of IDPs is therefore achieved by a distribution of conformational propensities. Interestingly, residual structures encoded in IDPs’ free state were shown to be precursors of conformational changes that may occur upon binding to a partner or upon self-assembly into oligomers or amyloid filaments.

**Fig. 2:**
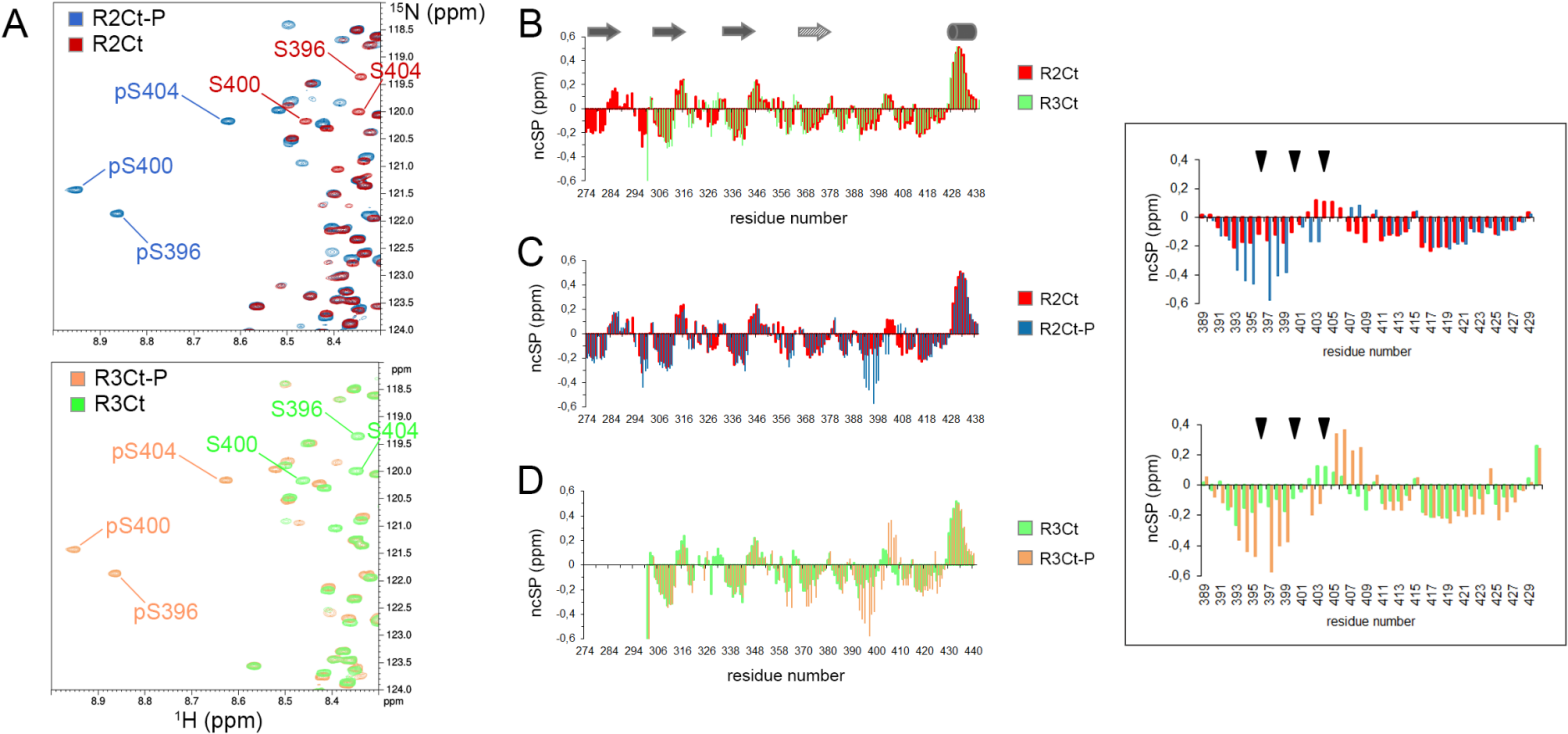
NMR characterization of the GSK3*β* phosphorylation pattern of tau R2Ct and R3Ct fragments, and conformational changes induced by GSK3*β* phosphorylation. (**A**) ^1^H-^15^N HSQC NMR spectra of tau fragments in their non-phosphorylated and GSK3*β* phosphorylated forms for R2Ct (red), R2Ct-P (blue), R3Ct (green) and R3Ct-P (orange) highlighting the homogenous triple phosphorylation of the PHF-1 epitope. (B, C, D) Neighbor-corrected secondary structure propensity (ncSP) scores calculated for R2Ct in its non-phosphorylated (red) or phosphorylated (blue) form, or R3Ct in its non-phosphorylated (green) or phosphorylated (orange) form. Comparisons of R2Ct and R3Ct (**B**), R2Ct and R2Ct-P (**C**), R3Ct and R3Ct-P (**D**) highlight conformational changes induced by phosphorylation, that are mainly localized around the PHF-1 site (inset, phosphorylation sites are indicated by triangles). The *β*-structure propensity found in each repeat is indicated by a arrow and the α-helix propensity in the extreme C-terminus by a cylinder.

In order to define the conformational propensity of R2Ct and R3Ct fragments, in their non-phosphorylated and GSK3*β-*phosphorylated forms, we produced them with uniform ^15^N, ^13^C isotopic labelling for NMR triple resonance backbone assignment. According to our previous characterization in full-length tau protein, GSK3*β* provided an homogenous triple phosphorylation of the C-terminal PHF-1 epitope at S396, S400 and S404 in both R2Ct and R3Ct fragments, as shown in ^1^H-^15^N HSQC spectra (Fig. 2A, Figures S1 and S2)^71^. CDK2/cyclin A phosphorylation of R2Ct and R3Ct fragments provided a homogenous phosphorylation of S404 and a lower phosphorylation level of S396, while sequential phosphorylation by CDK2/cyclin A and GSK3*β* resulted in the same phosphorylation pattern of the PHF-1 epitope as GSK3*β* alone, as previously described for full-length tau (Figures S1 and S2)^71^.

Based on the chemical shifts of H_N_, N, CO, Cα, Hα and Cβ nuclei, we have examined the conformational propensity of the different variants to scrutinize different conformations between both R2Ct and R3Ct fragments that would explain their distinct behavior in MT polymerization activity, and a differential effect of PHF-1 triple phosphorylation on the fragment conformation. ^13^C secondary chemical shifts of Cα and Cβ indicate a random coil conformation for all variants of tau fragments. As shown by neighbor-corrected Structure Propensity (ncSP) score^77^, the non-phosphorylated R2Ct and R3Ct tau fragments both adopt the same structure propensity profile (Fig. 2B). GSK3*β* phosphorylation induces a local conformational change within the PHF-1 sequence in both R2Ct and R3Ct fragments, however any remarkable difference in the phosphorylation-induced conformational changes has been noticed between R2Ct and R3Ct fragments (Fig. 2C, D). Consistent with previous observations in tau protein, each repeat domain has a propensity to adopt an extended conformation, including the R’ pseudo-repeat, and a 10-residue stretch of the extreme C-terminus (residues 427–437) shows a remarkable helical propensity^78^. The PHF-1 phosphorylation induces an additional extended conformation within the N-terminal region of the phosphorylation sites (residues 392–402) in both fragments as highlighted by ncSP scores (Fig. 2), secondary structure propensity (SSP)^79^ based on ^1^Hα, ^13^Cα and ^13^Cβ (Figure S4) and backbone torsion angles prediction from TALOS-N (Figure S5). In addition, the PHF-1 phosphorylated fragments have a slight propensity to adopt a turn conformation in a short sequence C-terminal to PHF-1 (pS404-L408). This tendency was previously shown to be induced by pS404 and exacerbated when other PTMs are present at either S396 and/or S400 in 20-mer peptides encompassing the (392–411) region^71^. Taken together, these data suggest that additional N- and C-terminal sequences in fragments do not affect the conformational changes induced by multiple phosphorylation, which do not propagate remotely.

In agreement with our observations in the PHF-1 epitope, phosphorylation of T231/S235 in AT180 epitope was previously shown to induce a N-cap stabilizing a α-helix in the C-terminal region of phosphorylation sites^75,80,81^. Furthermore, it has been proposed that an intramolecular salt bridge between phosphate and lysine or arginine side chain locks the peptide in a poly-proline II (PPII) conformation^82^. In the RT^231^PP sequence of tau, a salt bridge interaction between R230 and pT231 competes with an intermolecular salt bridge with tubulin^75^. Similarly, as evidenced by large perturbations of K395 NH resonance upon phosphorylation of PHF-1 epitope^71^, pS396 could lock K395 in a salt bridge preventing interactions of these residues with binding partners. This may partially explain the loss of function in tubulin polymerization.

### Seeding and cross-seeding of R2Ct and R3Ct aggregation by R2Ct and R3Ct seeds

**Fig. 3:**
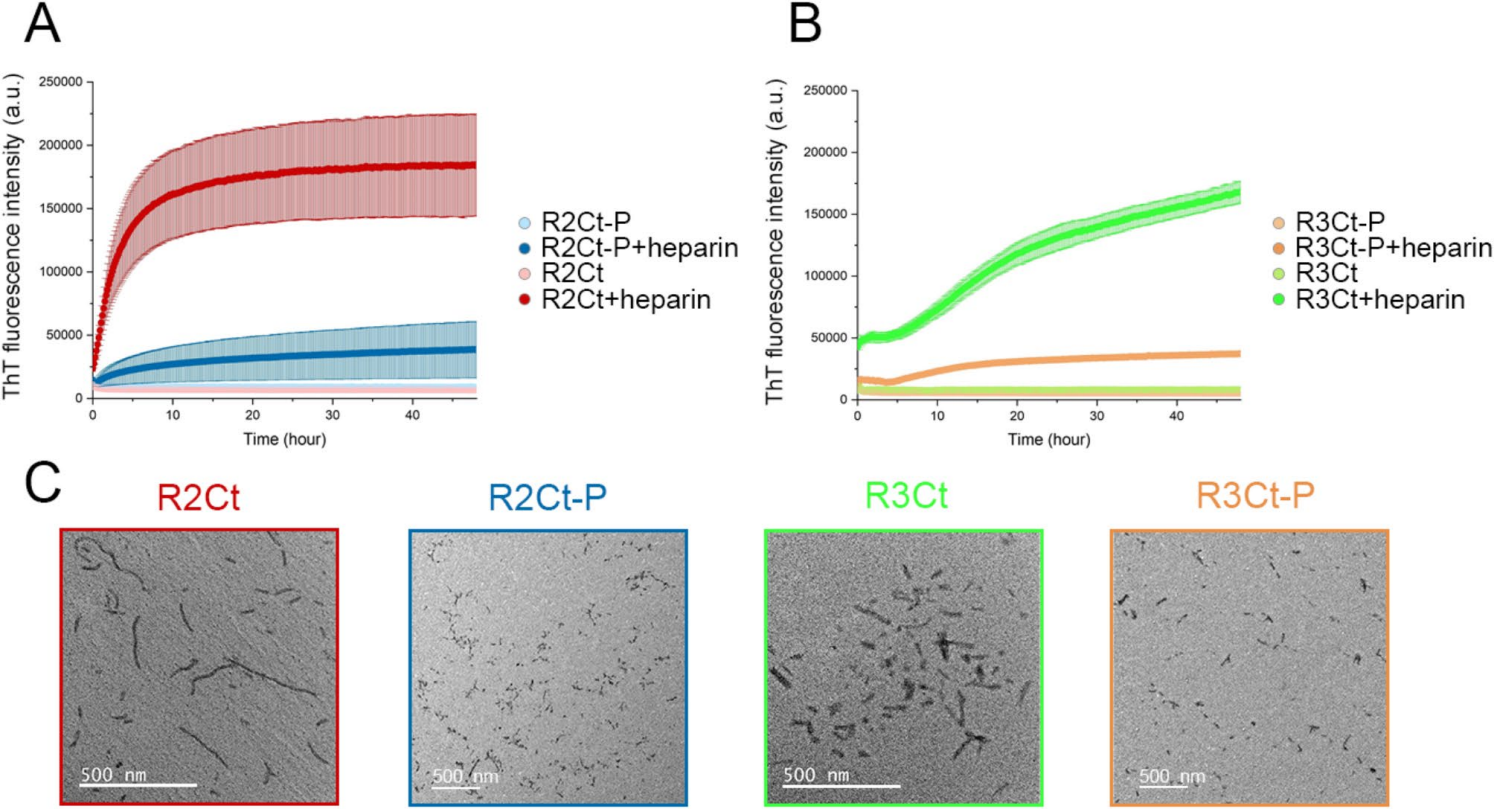
Assembly of tau R2Ct and R3Ct fragments and their GSK3*β* phosphorylated proteoforms into amyloid fibril structures induced by heparin. (**A**,**B**) Time-dependent aggregation monitored by increase of ThT fluorescence intensity for (**A**) tau R2Ct (red) and R2Ct-P (blue), and (**B**) R3Ct (green) and R3Ct-P (orange) fragments. Aggregation reactions of the different fragments without heparin are indicated with the same color code in light colors. (**C**) Negative staining TEM images showing fibrillar aggregates of R2Ct (red), R2Ct-P (blue), R3Ct (green) and R3Ct-P (orange).

R2Ct and R3Ct fragments and their GSK3*β*-phosphorylated proteoforms R2Ct-P and R3Ct-P were subjected to heparin-induced self-assembly for 48 h at 37 °C, as monitored by fluorescence of Thioflavin T (ThT) and TEM. Although tau filaments induced by heparin are structurally distinct from those extracted from AD brains^49–51^, no filaments were formed when fragments were incubated without heparin, as shown for similar non-phosphorylated fragments suggesting an inhibitory role of the C-terminus in fibrillation^53^. In the presence of heparin (at a tau: heparin ratio of 4:1), all fragments exhibit a time-dependent increase in ThT fluorescence suggesting the formation of amyloid-like structures (Fig. 3A, B). Specifically, the R2Ct fragment demonstrates a faster aggregation rate compared to R3Ct which starts aggregating after a lag-time of 5 h. After 48 h, R2Ct has reached a plateau while ThT intensity was still increasing for the R3Ct fragment. Moreover, both phosphorylated fragments, R2Ct-P and R3Ct-P, exhibit comparable aggregation rates than their non-phosphorylated counterparts although with much lower ThT signal intensities at the plateau (Fig. 3A, B). All fragments form filamentous aggregates, but of different length as shown by negative-staining TEM, with overall amounts in agreement with the relative ThT signals at end-point. R2Ct shows longer PHF-like fibrils than R3Ct, but with heterogenous length distribution, while R2Ct-P and R3Ct-P form aggregates of small size in which the PHF-like structure is not discernable (Fig. 3C).

**Fig. 4:**
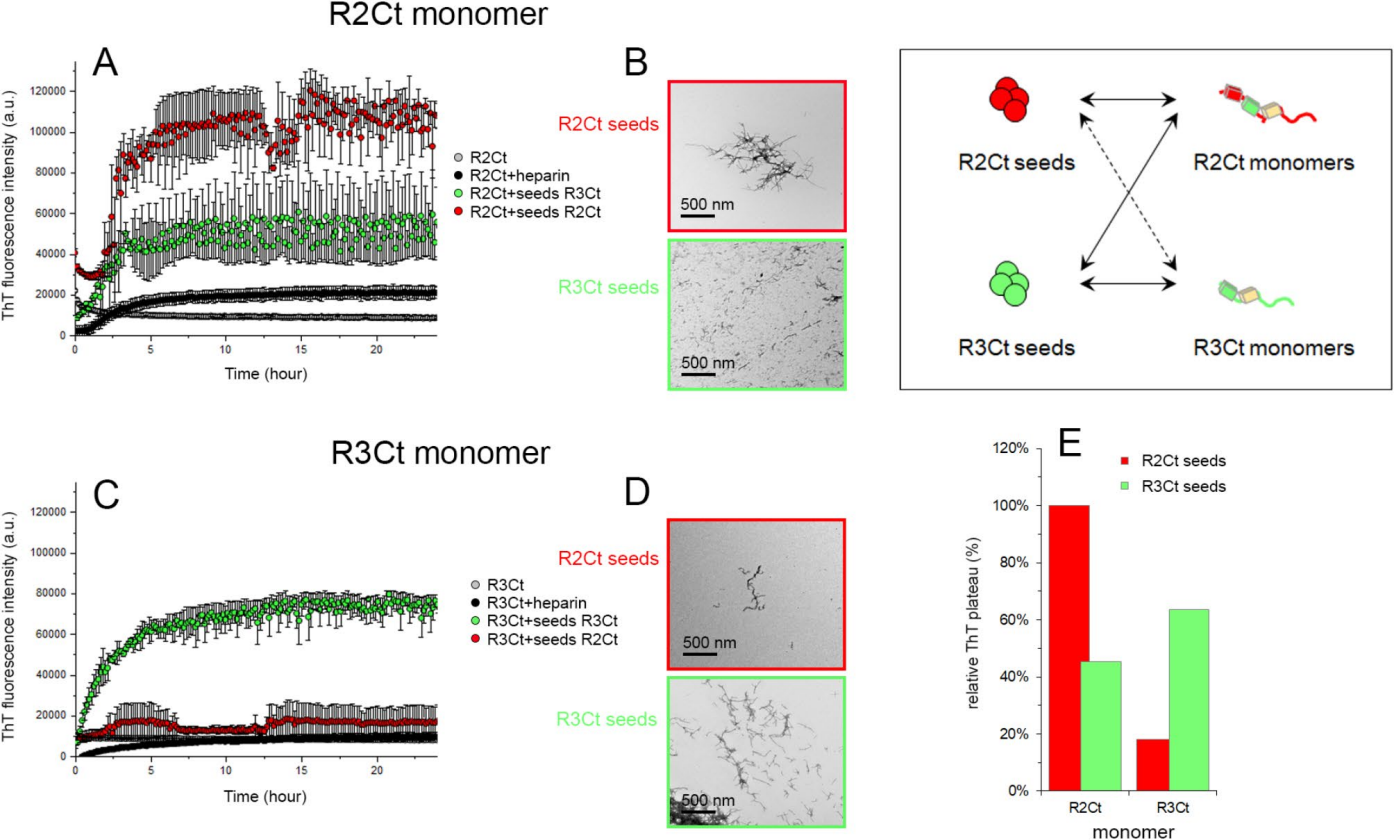
Seeding and cross-seeding activity of non-phosphorylated R2Ct and R3Ct seeds on non-phosphorylated R2Ct and R3Ct soluble monomers (inset). (**A**,**C**) Time-dependent seeding activity at 37 °C by 1 *µ*M of either R2Ct (red) or R3Ct (green) seeds on 25 *µ*M monomeric R2Ct (**A**) or R3Ct (**C**) as shown by changes of ThT fluorescence (three repeats represented as the mean ± SEM). Control of R2Ct or R3Ct fragment without heparin (grey) and at a high tau: heparin ratio (100:1) mimicking the heparin concentration afforded by dilution of seeds (black) are indicated. (**B**,**D**) Negative staining TEM images showing fibrillar aggregates induced by seeds of R2Ct (red) or R3Ct (green) on monomeric R2Ct (**B**) and R3Ct (**D**). (**E**) Graphical representation of the relative plateau of ThT signals normalized on R2Ct aggregation induced by R2Ct seeds.

Seeds were produced for both fragments in their non-phosphorylated and GSK3*β*-phosphorylated forms for 7 days at 37 °C using a higher tau: heparin ratio of 10:1 (Figure S6). According to the heparin concentration used here to generate seeds, the 10-fold dilution of seeds in subsequent seeding reactions with soluble monomers at 25 *µ*M leads to a heparin concentration of 0.1 *µ*M, which is below the critical concentration enabling association of tau fragments (Fig. 4A, C). Note that this value does not take into account the ratio of heparin embedded into the fibrils which is not determined. Seeds consisting of CDK2-phosphorylated fragments (R2Ct-P_C_ and R3Ct-P_C_) were produced to further evaluate the effect of an intermediate phosphorylation state.

We have evaluated first the seeding and cross-seeding properties of R2Ct and R3Ct seeds, phosphorylated or not by GSK3*β*, on aggregation of the non-phosphorylated or GSK3*β*-phosphorylated monomeric fragments at a final monomer concentration of 25 *µ*M for 1 *µ*M of seeds. In their non-phosphorylated form, R2Ct and R3Ct seeds have distinct seeding effects on the aggregation behavior of the soluble version of those fragments. R2Ct seeds induce aggregation of R2Ct fragment, as shown by a higher increase of ThT signal while having a limited impact on aggregation of R3Ct, suggesting a differential seeding activity on both protein variants. Conversely, R3Ct seeds exhibit similar seeding effects on aggregation of the R3Ct and R2Ct fragments (Fig. 4). These findings underscore that R2Ct and R3Ct seeds promote efficiently aggregation of their own soluble species. In addition, R3Ct seeds enable the cross-seeding of R2Ct aggregation while R2Ct seeds do not promote R3Ct aggregation (Fig. 4). This trend was previously observed for K18 and K19 fragments corresponding to the isolated MTBR domain of either the 4R and 3R isoforms, respectively, in which K19 seeds promote aggregation of K18, whereas K18 seeds do not seed aggregation of K19. It has been suggested using molecular dynamics that R2 and R3 repeats, as the most stable repeats in K18 and K19, respectively, are the respective catalytic centers for conformational selection in K18 and K19 fibril growth. Thus, R3 in K19 seeds is able to recruit similar R3 in K18 whereas K19 needs to cross a seeding barrier when fibril growth is induced by K18 seeds, since R2 is missing in K19^83^. In addition, R2 was shown by solid-state NMR to exhibit greater conformational plasticity than R3 in heparin-assembled tau fibrils, and this conformational adaptation was shown to regulate R2/R2 dimerization^84^. According to these findings, if R3 is the catalytic center of R3Ct fibrillization, it is able to template R3 conformational selection in both R2Ct and R3Ct monomers whereas R3Ct aggregation must overcome a cross-seeding barrier when R2Ct seeds template protein misfolding, involving R2 as the driver of conformational selection. These results suggest a differential role of fragments containing the R2 repeat or not in fibrillation of 3R and 4R isoforms as highlighted by the critical role of amino acid sequence spanning the R2-R3 junction to selectively seed aggregation of tau 4R isoforms and propagate aggregated intracellular tau^38^.

**Fig. 5:**
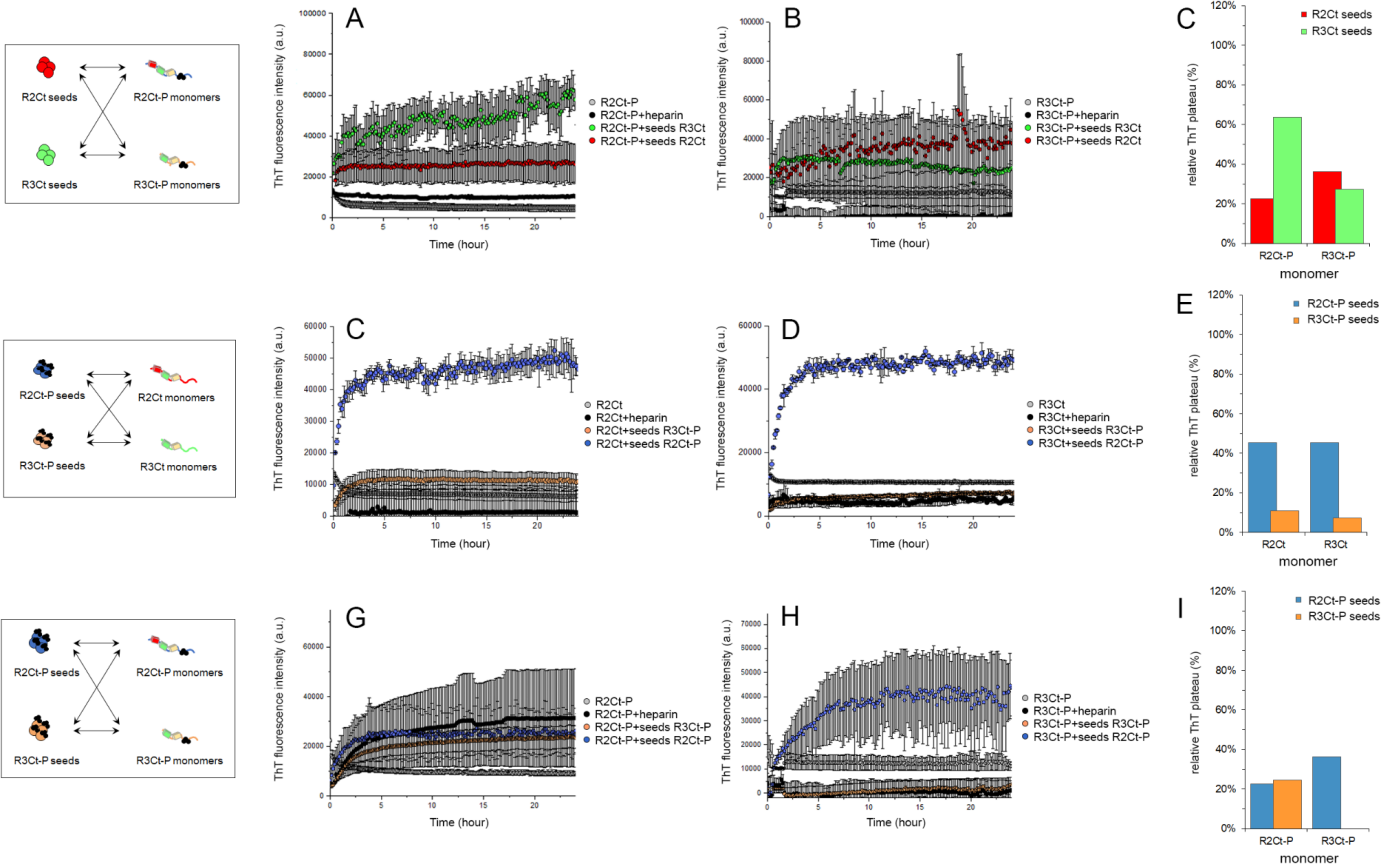
Cross-seeding activity of non-phosphorylated R2Ct (red) and R3Ct (green) seeds or phosphorylated R2Ct-P (blue) and R3Ct-P (orange) seeds measured by time-dependent changes of ThT fluorescence intensity showing the cross-seeding of phosphorylated R2Ct-P (**A**,**G**) and R3Ct-P (**B**,**H**) or non-phosphorylated R2Ct (**C**) and R3Ct (**D**) monomers at 25 µM with 1 µM of seeds. Aggregation of 25 µM tau fragments without heparin (grey) or with 0.1 µM heparin (black) are depicted as controls. The relative plateau of ThT signals is indicated for each condition after normalization on R2Ct aggregation induced by R2Ct seeds shown in Fig. 4, for the seeding of soluble R2Ct-P and R3Ct-P by non-phosphorylated (**C**) or phosphorylated (**I**) seeds, and the seeding of soluble R2Ct and R3Ct by phosphorylated seeds (**E**).

We then examined systematically each other cross-seeding combination involving the phosphorylated variants of R2Ct and R3Ct fragments either as seeds or monomers. The cross-seeding of non-phosphorylated R2Ct and R3Ct by phosphorylated seeds show comparable aggregation with R2Ct-P seeds and weak, if no aggregation, with R3Ct-P seeds as measured by increase of ThT fluorescence (Fig. 5C-E). Seeding of aggregation of phosphorylated monomers either with non-phosphorylated (Fig. 5A-C) or phosphorylated (Fig. 5G-I) R2Ct and R3Ct seeds shows lower overall efficiency than the seeding of aggregation of the non-phosphorylated monomers. R2Ct-P seeds seem to induce aggregation of all monomers more efficiently than R3Ct-P seeds, giving rise to a large number of short fibrils together with amorphous aggregates/oligomers (Fig. 5 and S7). In contrast, R3Ct-P seeds lead to the formation of few long fibrils from both non-phosphorylated R2Ct and R3Ct monomers (Figure S7). The same was observed with R3Ct seeding of R2Ct-P monomer. These results indicate that in the seeding conditions, the phosphorylated fragments have a lower capacity to aggregate than their non-phosphorylated variants, with R3Ct having an even lower capacity than R2Ct, similarly as previously observed for aggregation induced by heparin. Surprisingly, in contrast to what we have observed with seeding in non-phosphorylated conditions (non-phosphorylated monomers/seeds), the seeding in phosphorylated conditions (phosphorylated monomers/seeds) does not exhibit the same behavior, and the cross-seeding of aggregation, R3Ct-P by R2Ct-P seeds or R2Ct-P by R3Ct-P seeds, appears to be more efficient than homologous seeding (the same seed/monomer combination). However, the cross-seeding produces a high number of small fibrils and amorphous aggregates while seeding of R2Ct-P by homologous seeds rather produces few long fibrils.

The accessibility of the aggregation nuclei (PHF6 and PHF6* sequences) is known to be a critical factor in discriminating between seed-competent and inert monomers while the seeding capacity encoded in the monomer can propagate strains in neuronal cells^22,85^. Thus, the higher cross-seeding capacity of R2Ct-P over R2Ct seeds could originate from changes in R2 repeat accessibility upon PHF-1 hyperphosphorylation, shifting the center for conformational selection to another region that favor cross-seeding of the R3Ct fragment. We have shown that PHF-1 phosphorylation induces a propension of the 392–402 sequence to adopt a rigid β-structure in the soluble, monomeric state, that could be stabilized within the seeds, potentially altering the accessibility of R2. Together, these results suggest that PHF-1 hyperphosphorylation of seeds may decrease the cross-seeding barrier and/or may change the catalytic center for conformational selection.

### Seeding of 2N4R Tau aggregation by R2Ct and R3Ct seeds

We then investigated the seeding properties of non-phosphorylated and phosphorylated fragments to induce aggregation of full-length tau containing or not the aggregation-prone P301L mutation. First, the aggregation capacity of 2N4R tau proteins was assessed by heparin induction, and both tau and tau-P301L mutant show comparable kinetics with elongation rate constants of 0.4 and 0.2 h^-1^, respectively, but tau-P301L reached a plateau two-fold higher than tau and both formed PHF-like fibrils of various lengths (Figure S8).

**Fig. 6:**
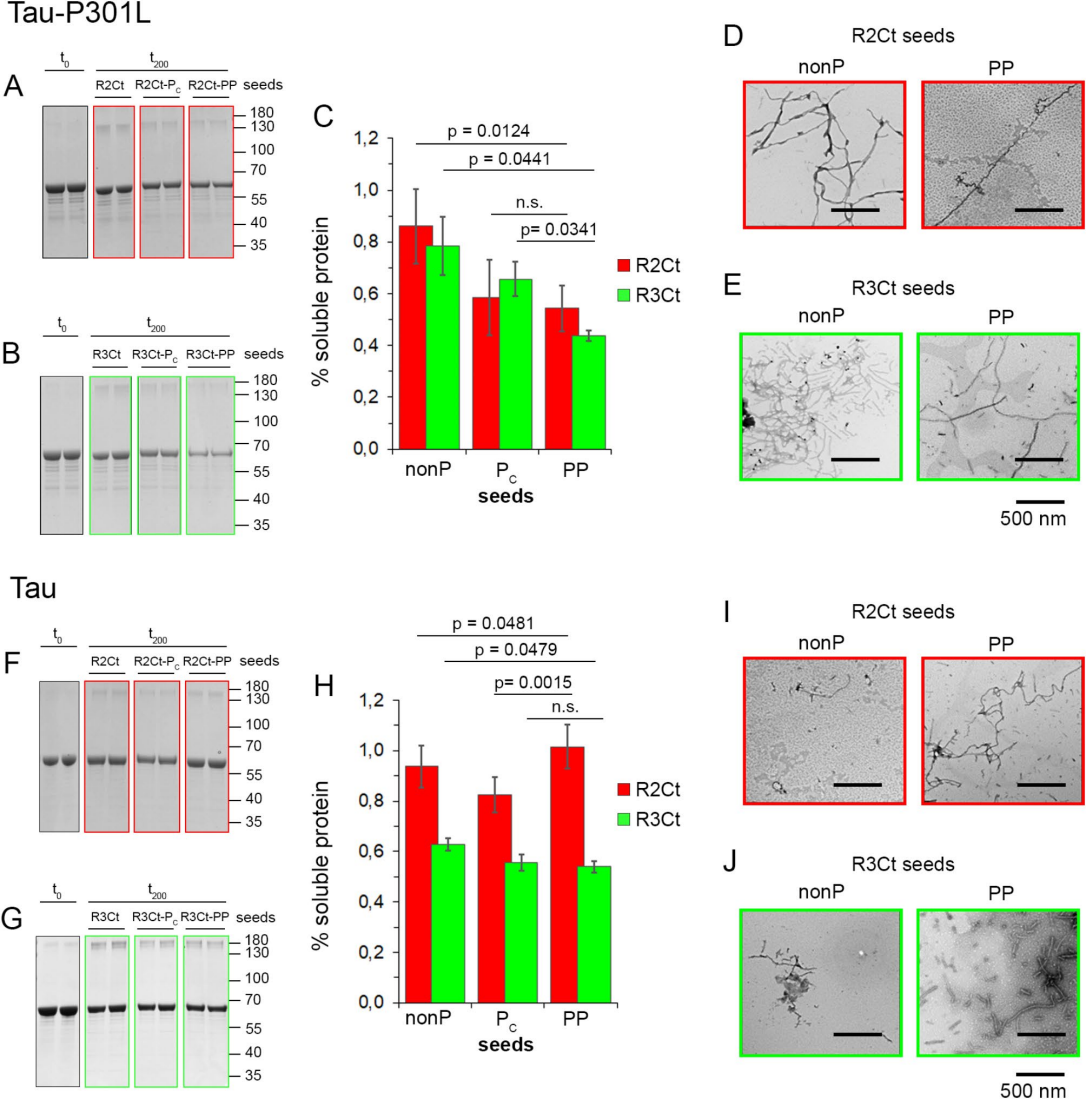
Cross-seeding activity of non-phosphorylated and phosphorylated R2Ct (red) or R3Ct (green) seeds on full-length tau 2N4R soluble monomers, either tau-P301L mutant (**A**–**E**) or tau without the aggregation-prone mutation (**F**–**J**). The seeds are prepared from R2Ct or R3Ct fragments without phosphorylation (nonP) or after phosphorylation by CDK2 (R2Ct-P_C_ or R3Ct-P_C_) or sequential phosphorylation by CDK2 and GSK3β (R2Ct-PP or R3Ct-PP). The aggregation of full-length tau is studied in a sedimentation assay upon treatment with R2Ct (**A**,**F**) or R3Ct seeds (**B**,**G**), shown as duplicates of each condition delineated by colored boxes. The extent of tau-P301L (**C**) or tau (**H**) aggregation is measured as the percentage of soluble protein after 200 h incubation at 37 °C (t_200_) with R2Ct (red) or R3Ct (green) seeds relative to the initial protein concentration before incubation (t_0_). Results are represented as the mean of technical replicates ± SEM (*n* = 2). Unpaired two-tailed Student’s t tests were used for statistical analysis. Negative staining TEM images showing fibrillar aggregates induced by seeds of R2Ct (red) or R3Ct (green), either non-phosphorylated (nonP) or phosphorylated by CDK2/GSK3β (PP), on tau-P301L (**D**,**E**) or tau (**I**,**J**). Full gel images for panels A, B, F and G are shown as additional materials in Figure S12.

Then, we performed aggregation of 2N4R tau proteins upon seeding with non-phosphorylated or CDK2/GSK3β-phosphorylated R2Ct and R3Ct seeds (R2Ct-PP and R3Ct-PP), or without seeds as controls. Additionally, fragments only phosphorylated by CDK2 were used as seeds with intermediate phosphorylation level (R2Ct-P_C_ and R3Ct-P_C_), mimicking a more physiological phosphorylation as opposed to GSK3β phosphorylation alone or after priming by CDK2, leading to homogenous PHF-1 hyperphosphorylation (Fig. 2, Figures S1 and S2). Kinetics of aggregation was followed by changes of ThT fluorescence (Figure S9). However, the initial steps of nucleation/elongation when tau aggregation is induced by seeds appear to be difficult to detect under some conditions, starting at a high intensity of ThT signal without any changes over time, preventing kinetics analyses. Although high initial ThT signals may arise from binding of preformed seeds to ThT, this process alone is not sufficient to produce the signal levels detected in the very first stages, considering the steady-state ThT signals of R2Ct/R3Ct aggregation reactions (Fig. 3) and the tenfold dilution of seeds in cross-seeding reactions. Such initial ThT signal may also account for a significant acceleration of the nucleation phase in the presence of preformed seeds. Taking advantage of the difference in molecular weight between the fragments used as seeds and 2N4R tau monomers, the seeding of aggregation was additionally followed by sedimentation assays and negative-staining TEM at end-point (200 h) (Fig. 6). It should be noticed that loss of soluble tau or tau-P301L monomers between t_0_ and t_200_ in the sedimentation assay may account for the formation of different species ranging from high molecular-weight (HMW) soluble oligomers (as shown by HMW bands in Fig. 6 and Figure S12) or insoluble oligomers, amorphous aggregates to *bona fide* fibrils observed by TEM.

As previously described^24,86^, tau-P301L mutant is more prone to aggregation than tau when seeded by preformed R2Ct or R3Ct seeds. R3Ct is more efficient than R2Ct to seed tau aggregation (without aggregation-prone mutation), and its seeding activity is independent of the phosphorylation state (Fig. 6F-H). In contrast, seeding of tau-P301L aggregation with R2Ct and R3Ct seeds is comparable, but depends on the PHF-1 phosphorylation state with phosphorylated R2Ct-PP and R3Ct-PP seeds exhibiting stronger seeding activity than CDK2-phosphorylated and non-phosphorylated seeds, which is more predominant for R3Ct (Fig. 6A-C). TEM imaging show the presence of PHF-like filaments longer than the fibrils or aggregates used as seeds (Figs. 3 and 6). The loss of soluble protein after 200 h-incubation at 37 °C with seeds observed in sedimentation assays is not systematically correlated with the number of fibrils suggesting that insoluble species other than PHF-like fibrils may be formed under different seeding conditions. In addition, immunoelectron microscopy of the fibrils using tau HT-7 monoclonal antibody, which selectively stains 2N4R tau, and nanogold labelling assessed the incorporation of full-length tau monomers into fibrils (Figure S10).

Although R2Ct contain a greater proportion of 2N4R tau isoform than R3Ct, the seeding barrier for R3Ct seeds to promote tau aggregation appears to be lower than for R2Ct seeds, supporting the previous observation that R2 undergoes a conformational dynamics into fibrils that has been shown to regulate fibrillar assembly^84^. However, for the tau-P301L mutant, the seeding barrier of fibrillar assembly is comparable for R2Ct and R3Ct seeds that both contain the P301 site without mutation, and phosphorylation of seeds decreases the cross-seeding barrier with the tau-P301L monomer. Localized in R2 near the R2-R3 junction, the P301L mutation was proposed to facilitate aggregation by exposing of the aggregation-prone PHF6 sequence through the disruption of a fold that prevents the formation of a β-sheet^22,87,88^. Therefore, it is likely that this mutation in itself reduces the seeding barrier, rendering both R2Ct and R3Ct equally capable of promoting fibrillar assembly of tau-P301L. Furthermore, we found that tau-P301L aggregation is facilitated by seed phosphorylation suggesting that PHF-1 phosphorylation, by inducing a conformational change, alters the seeding barrier and/or shifts conformational selection to the PHF-1 sequence, which has a similar trend to fold into a β-sheet conformation as R2 and R3 repeats (Fig. 2, Figures S4 and S5). In contrast, the aggregation of tau without the aggregation-prone mutation is not responsive to PHF-1 phosphorylation of seeds, and therefore likely driven by the R3 repeat. This has also been shown in the truncated tau (297–391) that self-assembles into AD filaments in vitro in which the region comprising residues 302–316 forms an ordered core in the first filament intermediates^67^.

Deciphering the spreading of tau pathology associated to neurodegenerative disorders involves the identification of specific tau species that are transmitted neuron-to-neuron as well as the underlying mechanisms of release and uptake of pathological species. These questions are still into focus and the characterization of tau seeds remains a challenging task. Tau PTMs have been highlighted as molecular biomarkers of diseases and were recently associated with the clinical heterogeneity of AD and severity of tau pathology in AD patients. Notably, some phosphorylation sites (S262, T231, S235) were positively correlated with an increased seeding activity in the soluble, HMW tau fraction isolated from AD brains, which was not the case for PHF-1 phosphorylation (S396, S404) that exhibited a negative correlation with the seeding activity^56^. In our study, seeds from R2Ct/R3Ct fragments, hyperphosphorylated at the unique PHF-1 site by GSK3*β* or non-phosphorylated, contain not only soluble HMW species but also insoluble aggregates and twisted filaments. These seeds were able to promote assembly of different tau variants with different cross-seeding efficiencies, depending on the combination of the seed phosphorylation state and the monomer.

## Conclusion

Aggregation takes its origin in protein misfolding and the resulting fibrils can act as templates either for monomer misfolding and incorporation during the fibril elongation step or in a surface-catalyzed secondary nucleation to generate new fibrils^89–91^. Seeding of aggregation is therefore suggested as one of the mechanisms that contributes to fibrillar assembly and propagation of tau pathology where seeds act as templates to support the formation of new fibrils. Seed-competent species can be as simple as single misfolded monomers, but can also involve small to large oligomer assemblies or aggregates comprising a large panel of tau proteoforms which contain various types and sites of PTMs, not restricted to phosphorylation, and truncated variants^22,48,92–96^. However, the molecular mechanism of tau aggregation and spreading as well as the species involved in the seeding of pathological tau assembly still remain open questions.

Here, we have focused our study on R2Ct and R3Ct tau fragments spanning the entire C-terminal domain starting from either the R2 or R3 repeats. Therefore, these fragments may be proteolytically produced from both 4R and 3R isoforms for R3Ct, or only 4R isoforms for R2Ct. We have shown that R2Ct and R3Ct have a reduced or complete loss of tubulin polymerization activity, respectively. GSK3*β* phosphorylation, by providing triple phosphorylation of the PHF-1 epitope, prevents tubulin polymerization and promotes a local conformational change resulting in an additional propensity to fold into an extended *β*-sheet conformation. This extends over a sequence encompassing part of the PHF-1 motif (residues 392–402) in both fragments. Such a conformational change may be stabilized in the seeds and could alter the accessibility of PHF6* and PHF6 in the R2 and R3 repeats, respectively, that are critical sequences in the nucleation of aggregation.

Given the loss of function in MT polymerization, we investigated the pathological function of these fragments, phosphorylated or not, in the seeding of aggregation. Fragment seeds induced by heparin showed a capacity to seed aggregation of the soluble version of those fragments under conditions that are dependent on the monomer/seed combination and their phosphorylation state. We have found that R3Ct seeds are able to template seeding of both R2Ct and R3Ct monomers while R2Ct seeds only promote homologous seeding, suggesting that a cross-seeding barrier exists to induce aggregation of R3Ct by R2Ct seeds related to the presence of the R2 repeat or not.

Our study has also highlighted that PHF-1 hyperphosphorylation decreases the cross-seeding barrier for R2Ct to induce tau-P301L aggregation and/or change the conformational selection to recruit monomers into seeds. In contrast, it has little impact on the aggregation of tau without the aggregation-prone mutation, that is likely driven by the R3 repeat. Our findings indicate that the seeding of aggregation is an intricate cooperative mechanism that involved a complex interplay between the efficiency of seeds to template conformational changes and the aggregation capacity of the monomers.

## Methods

### Tau protein and fragment expression and purification

The DNA sequences encoding tau R2Ct (272–441) and R3Ct (300–441) (Figure S11) were inserted into the pET15b plasmid into *Nco*I and *BamH*I restriction sites. Then, the plasmids were introduced into *E. coli* BL21(DE3) strain. For protein expression using NMR-active isotopes, a large-scale bacterial culture was started from a 20 ml culture by inoculating 1 L of M9 minimal medium, containing 1 g/L ^15^NH_4_Cl, 2 g/L ^13^C_6_-D-glucose, 0.5 g/L ^15^N,^13^C-ISOGRO^®^(Sigma-Aldrich), 1 mM MgSO_4_, 100 µM CaCl_2_, and 1 mg/L ampicillin. The bacteria were grown at 37 °C for approximately 4 h to reach an optical density at 600 nm (OD_600_) of 0.9-1.0, protein expression was induced by the addition of 1 mM isopropyl-*β*-D-thiogalactopyranoside (IPTG) and the culture was continued for 3 h at 37 °C. The bacterial pellet was collected following a 20 min-centrifugation at 6,000 · *g* and 4 °C, resuspended in 20 mL of PBS buffer and centrifuged at 6,000 · * g* and 4 °C for 20 min, then stored at -20 °C. For purification, the bacterial pellet was thawed and suspended in 20 mL of extraction buffer composed of 50 mM sodium phosphate buffer pH 6.5, 1 mM EDTA, 2 mM dithiothreitol (DTT) supplemented with EDTA-free protease inhibitor cocktail (cOmplete, Roche) and 3,000 U DNase I. The cells were lysed using a high-pressure homogenizer at 4 °C. The lysate was centrifuged for 30 min at 30,000 · *g* and 4 °C to remove insoluble material, and the soluble extract was then heated for 15 min at 80 °C. A subsequent round of centrifugation removed precipitated proteins and the supernatant was loaded onto a 5 mL HiTrap SFF column (Cytiva) equilibrated with loading buffer (50 mM sodium phosphate buffer pH 6.5, 1 mM EDTA). The column was extensively washed with loading buffer, then proteins were eluted by applying a linear gradient of elution buffer (50 mM sodium phosphate buffer pH 6.5, 1 mM EDTA, 1 M NaCl). The elution fractions were analyzed by MALDI-TOF MS and SDS-PAGE. Fractions containing tau fragments were pooled, loaded on a reverse-phase chromatography column (Zorbax 300SB C8 9.4 ⋅ 250 mm, Agilent) equilibrated with loading buffer (5% acetonitrile, 0.1% TFA), and eluted at 5 ml/min with a linear gradient of elution buffer (80% acetonitrile, 0.1% TFA) from 10 to 60% elution buffer in 50 min. Fractions were analyzed by MALDI-TOF MS, and those containing tau fragments were lyophilized. The lyophilized proteins were resuspended in 50 mM ammonium bicarbonate buffer and loaded on a desalting column (HiTrap Desalting 5 ml, Cytiva) equilibrated with the same buffer. Eluted proteins were then lyophilized and stored at -20 °C for further use. 2N4R tau proteins with S262A or P301L point mutation, or the tau-PHF1 mutant containing mutations at several phosphorylation sites^71^, were produced following the same procedure^46,74^.

### GSK3β and CDK2/cyclin A expression and purification

The DNA sequence of human GSK3*β* isoform was synthesized with codon optimization for *E. coli* expression, then inserted into the pET21a plasmid into *Nde*I and *Xho*I restriction sites encoding a C-terminal poly-histidine sequence. Then, *E. coli* BL21(DE3) bacteria were transformed with the plasmid for protein expression. The culture was performed as described for tau protein and fragments, except that the protein was produced in 1 L of LB medium and induction of protein expression was made at 20 °C for 16 h with addition of 1 mM IPTG. The bacterial pellet was then resuspended in extraction buffer containing 50 mM sodium phosphate buffer pH 7.8, 300 mM NaCl, 10 mM imidazole, 0.5% NP-40, supplemented with EDTA-free protease inhibitor cocktail (cOmplete, Roche) and 3,000 U DNase I. The soluble extract was prepared as described for tau proteins, then proteins were purified on a nickel-nitrilotriacetic acid (Ni-NTA) chromatography column (HisTrap HP 1 ml, Cytiva) equilibrated with 50 mM sodium phosphate buffer pH 7.8, 300 mM NaCl, 10 mM imidazole. Then, the proteins were eluted at 2 ml/min with a linear gradient of elution buffer (50 mM sodium phosphate buffer pH 7.8, 300 mM NaCl, 250 mM imidazole) from 0 to 100% in 10 min. The homogenous fractions were pooled and buffer exchanged into phosphorylation buffer (50 mM Hepes.KOH, pH 7.8, 50 mM NaCl, 12.5 mM MgCl_2_, 1 mM EDTA, 1 mM DTT) to obtain about 5 mg per liter of culture at approximately 1 mg/ml (15 *µ*M). GSK3*β* kinase activity was assessed on a 20-mer tau peptide primed at pS404 as described^97^.

The CDK2/cyclin A complex is produced in *E. coli* BL21(DE3) strain as described^97^. Briefly, CDK2 is co-expressed as a GST-fusion protein with the yeast CIV1 kinase at 16 °C overnight, and cyclin A is expressed independently at 16 °C overnight. Both soluble extracts are pooled and purified on glutathione sepharose beads, then eluted by cleavage of the GST tag by the PreScission Protease. 10 mg of the CDK2/cyclin A complex is typically obtained from 1 L of culture (of each protein) at a concentration of about 0.5 mg/ml (7.7 µM).

### CDK2/cyclin A and GSK3β phosphorylation of Tau fragments

For the phosphorylation of tau fragments, a mixture was prepared by combining 100 *µ*M recombinant ^15^N/^13^C-labeled fragment with either 50 *µ*L GSK3*β*-His_6_ or CDK2/cyclin A in a final volume of 500 *µ*L. The phosphorylation was performed at 25 °C in the presence of 1 mM ATP (or without ATP as a control in a smaller scale reaction) and complemented with protease inhibitors. A fraction of tau fragments phosphorylated first by CDK2/cyclin A was subsequently phosphorylated by GSK3*β*. Post-incubation, the samples were heated at 80 °C for 15 minutes, followed by centrifugation at 15,000 · *g* for 15 min. The supernatants were pooled, and subjected to centrifugation at 4,000 · *g* for 15 minutes at 4 °C to remove a residual precipitate. Subsequently, phosphorylation and control reactions were purified through reverse-phase chromatography on a C8 semi-preparative column as described for purification of tau protein and fragments. The eluted fractions were lyophilized, dissolved in 200 *µ*L of 5% acetonitrile, 0.1% TFA solution, and analyzed by MALDI-ToF MS^97^. Homogeneous fractions containing either GSK3*β*-phosphorylated (R2Ct-P and R3Ct-P), CDK2-phosphorylated (R2Ct-P_C_ and R3Ct-P_C_) or sequentially CDK2/cyclin A-GSK3*β* phosphorylated (R2Ct-PP and R3Ct-PP) tau fragments were desalted in 50 mM ammonium bicarbonate, and stored lyophilized at -20 °C until further use.

The phosphorylation reactions were controlled for their phosphorylation levels and patterns by MALDI-TOF MS and high-resolution NMR, respectively. Phosphorylation by CDK2 results in 89% and 44% phosphorylation of S404 and S396, respectively, as estimated by relative peak intensities on NMR^1^H-^15^N HSQC spectra. Note that the double phosphorylation pS396/pS404 occurs statistically, but the signals on the NMR spectra cannot be used to assess this phosphorylation state because the two residues are too far away from each other in the protein sequence. Combination of CDK2 and GSK3β, or GSK3β alone, result in homogenous phosphorylation on each S396, S400 and S404 site as evidenced by the complete disappearance of the peak corresponding to their respective non-phosphorylated form (Figures S1 and S2). These site-specific phosphorylation levels are in good agreement with the overall phosphorylation levels measured by MS (Figure S1).

### NMR spectroscopy

#### Sample preparation

For NMR experiments, ^15^N, ^13^C-labeled R2Ct (350 *µ*M), R2Ct-P (350 *µ*M), R3Ct (300 *µ*M) and R3Ct-P (350 *µ*M) NMR samples were prepared in 300 *µ*L of 50 mM phosphate buffer pH 6.6, 50 mM NaCl, 2.5 mM EDTA, 1 mM DTT and 1/10 (v/v) D_2_O. For chemical shift referencing, 1 mM sodium 3-trimethylsilyl-3,3′,2,2′-d_4_-propionate was added to the sample and served as the ^1^H reference, with ^15^N and ^13^C chemical shifts indirectly referenced based on ^1^H chemical shifts.

### Data acquisition and processing

3 mm-NMR Shigemi tubes were filled with tau samples for acquisition of 2D and 3D experiments at 293 K. R2Ct and R2Ct-P experiments were conducted at 293 K using a 900 MHz Bruker Avance spectrometer equipped with a triple- resonance cryogenic probe head. For R3Ct and R3Ct-P, a 600 MHz Bruker Avance III HD spectrometer with a CPQCI cryoprobe was employed.

Classical backbone assignments were established through various 3D experiments, including HNCACB, HNCO, HNcoCACB, HNcaCO, HncaNNH, HncacoNNH, hNcaNNH, and hNcacoNNH. Simultaneously, proline resonances were assigned using carbon-detected 3D hCACON and HCAN experiments, along with 2D NCO and CACO experiments. All 3D spectra were acquired using non-uniform sampling. Data acquisition and processing were carried out using Bruker Topspin 3.5. Following this, NMRFAM-Sparky was employed for NMR data analysis, and the results were cross-validated using the I-PINE web server^98,99^.

### Tubulin polymerization assay

The tubulin polymerization assay was conducted using the fluorescence-based tubulin polymerization assay (Cytoskeleton, Inc. Cat. # BK011P). Each fragment, phosphorylated or not, was prepared at a 10-fold concentration (200 *µ*M) in a solution containing 80 mM PIPES pH 6.9, 0.5 mM EGTA, and 2.0 mM MgCl_2_. To reconstitute the tubulin polymerization assay, 1 mM GTP, 2 mg/mL of tubulin (porcine tubulin, prepared according to the kit recommendations) and 20 *µ*M of tau fragments are dissolved in 1.5 mL of polymerization buffer without glycerol for detecting polymerization enhancing compounds. Subsequently, 55 *µ*L of the reaction mixture containing an equimolar ratio of tau: tubulin at 20 *µ*M was dispensed into a 96-well plate (Corning Costar, Cat. # 3686). In addition, fragments and 2N4R tau proteins as controls were also analyzed at 10 µM with tubulin dimer at 20 µM in polymerization buffer supplemented with 5% glycerol that are conditions improving tubulin polymerization. Kinetics of tubulin polymerization into MTs were monitored at 37 °C using the increase in fluorescence emission at 460 nm over a 60-minute period at 37 °C on a plate reader (PHERAStar, BMG LABTECH GmbH, Ortenberg, Germany). Three replicates were performed for each condition.

### In vitro heparin-induced aggregation

To generate fibrils of R2Ct and R3Ct fragments, and their phosphorylated variants, 10 *µ*M of fragments were incubated in aggregation buffer containing 50 mM MES pH 6.9, 2.5 mM EDTA, 30 mM NaCl and 0.33 mM DTT supplemented with 2.5 *µ*M of heparin in a final volume of 1 mL. In parallel, monitoring of fibril formation was performed by addition of 50 *µ*M Thioflavin T (ThT) fluorescent probe in 100 *µ*L of the same reaction mixture to track the aggregation kinetics (PHERAStar, BMG LABTECH GmbH, Ortenberg, Germany). The aggregation reactions were then incubated for 3 to 5 days at 37 °C. The seeds are subsequently collected for seeding experiments and negative-staining transmission electron microscopy.

### Seeding and cross-seeding assays

Seeds were prepared in the same aggregation buffer with 10 µM of tau fragments (R2Ct, R2Ct-P, R3Ct, or R3Ct-P) in the presence of 1 *µ*M of heparin for 7 days at 37 °C. Seeding and cross-seeding assays closely mirror the aggregation reaction used to produce seeds, except that heparin was substituted by the seeds. Seeds from aggregation reactions were diluted at a final concentration of 1 *µ*M in the seeding reactions in the presence of 25 *µ*M of soluble, monomeric 2N4R tau proteins or fragments. Accordingly, heparin included within the seeds is also diluted, ultimately yielding a final concentration of 0.1 *µ*M. A negative control conducted without seeds in the presence of 0.1 *µ*M of heparin showed that this concentration of heparin alone is insufficient to stimulate the fibril formation in the absence of seeds. To prepare the seeds, the R2Ct and R3Ct samples in their non-phosphorylated and phosphorylated forms have been dosed to equal concentration prior to the aggregation reactions (and checked for integrity and homogeneity by MS and NMR). We have considered as “seeds” the products of the aggregation reactions without fractionation (e.g., into soluble and insoluble fractions). Thus, the seeding species (HMW soluble oligomers, insoluble species or fibrils) may differ in the different conditions and occur in different ratios (Figure S6).

Seeding reactions of tau 2N4R isoform were performed on tau-P301L and tau without aggregation-prone mutation (tau-S262A) induced by R2Ct or R3Ct seeds either in their non-phosphorylated form or phosphorylated forms provided by CDK2 alone or a combination of CDK2 and GSK3 kinase activities. Aggregation induced by seeds were followed by time-dependent changes in ThT fluorescence in duplicates. Negative-staining TEM imaging and sedimentation assay measuring the decrease in soluble monomer concentration were performed at the aggregation end-point (200 h). Protein bands on SDS-PAGE were quantified by densitometry (ImageQuantTL 10.0.261 software, Cytiva) and the extent of protein aggregation was determined by the ratio of protein amount after 200 h-incubation at 37 °C with seeds (t_200_) on the initial amount before incubation with seeds (t_0_). Each sample was analyzed in duplicates.

### Transmission electron microscopy

Transmission electron microscopy imaging was performed on aggregation reactions by negative-staining (JEOL JEM-2100 microscope at 200 kV). 3 *µ*L from the aggregation reactions were applied to 400-mesh hexagonal nickel grids for a 30 s. Then, the solution was carefully removed using a filter paper, and the grids were washed three times with ultrapure water. 10 *µ*L of 2% uranyl acetate was applied for 10 s for negative staining, then solution was removed using a filter paper. The grids were subjected to a 60 s re-staining with 2% uranyl acetate, followed by solution removal.

Immunolabelling with 6 nm-nanogold particles was performed with HT-7 monoclonal antibody that selectively recognizes 2N4R tau isoform, not fragments from seeds (antigen 159-PPGQK-163, 2N4R tau numbering). For fibril immunogold labelling, 5 µL of the aggregation sample was applied on 400-mesh hexagonal nickel grids for 1 min. After sample removal and three washes of fresh PBS buffer, the grids were incubated with 5% BSA (w/v) in PBS for 30 min. The grids were incubated with the primary HT-7 antibody (1:100) in 1% BSA for 30 min followed by five washes in PBS, then incubated with the gold-labelled secondary antibody (6 nm-gold donkey IgG, 1:40) in 1% BSA for 30 min, followed by five washes in PBS and ten washes in water before staining with uranyl acetate 2% (w/v) for 1 min. TEM imaging was performed using a HITACHI H7500 microscope at 80 kV.

## Electronic supplementary material

Below is the link to the electronic supplementary material.

Supplementary Material 1

## Data Availability

Chemical shift assignments of R2Ct and R2Ct-P fragments for ^1^H, ^15^N, and ^13^C backbone and partial sidechain NMR resonances were deposited in the BioMagResBank with entry number 52309 and 52401, respectively. In total, 96% of backbone resonances were assigned including N, HN, H*α*, C*α*, CO and partial side chain (C*β*) resonances (397 resonances).
