# Supplementary material for: Effect of PHF-1 hyperphosphorylation on the seeding activity of C-terminal Tau fragments
Source: Sci Rep. 2025 Mar 22;15:9975. doi: 10.1038/s41598-025-91867-3 (PMC11929799; doi:10.1038/s41598-025-91867-3)
Supplement: Supplementary file 1 — Supplementary Material 1 [file 41598_2025_91867_MOESM1_ESM.docx]

# Supplementary Information

# Effect of PHF-1 hyperphosphorylation on the seeding activity of C-terminal tau fragments

Léa El Hajjar,^1,2^ Emmanuelle Boll,^1,2^ François-Xavier Cantrelle,^1,2^ Clarisse Bridot,^1,2^ Isabelle Landrieu,^1,2^ and Caroline Smet-Nocca^1,2*^.

1 Univ. Lille, Inserm, CHU Lille, Institut Pasteur de Lille, U1167 - RID-AGE - Risk Factors and Molecular Determinants of Aging-Related Diseases, F-59000 Lille, France

2 CNRS EMR9002 Integrative Structural Biology F-59000 Lille, France

*Corresponding Author: Caroline Smet-Nocca – Inserm U1167/ Institut Pasteur de Lille, 1 rue Professeur Calmette, BP245, 59019 Lille, France; orcid.org/0000-0002-9793-0882; Email: [**caroline.smet-nocca@univ-lille.fr**](mailto:caroline.smet-nocca@univ-lille.fr)

**Figure S1**

A


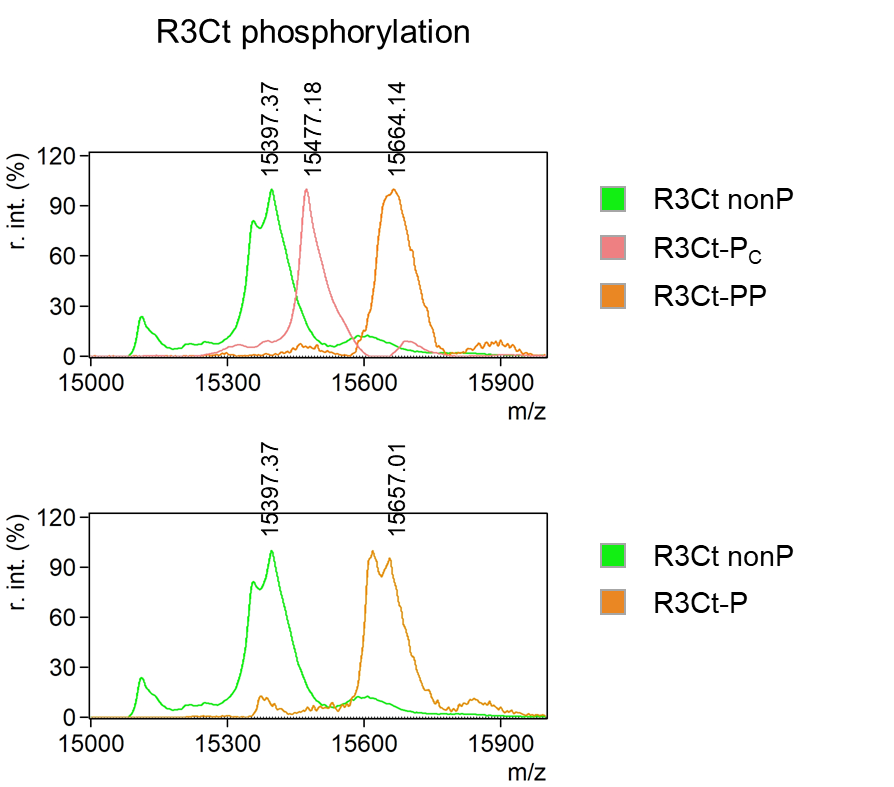


B


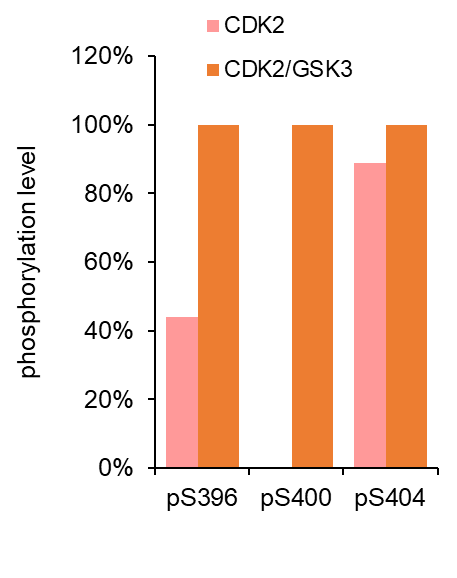


C


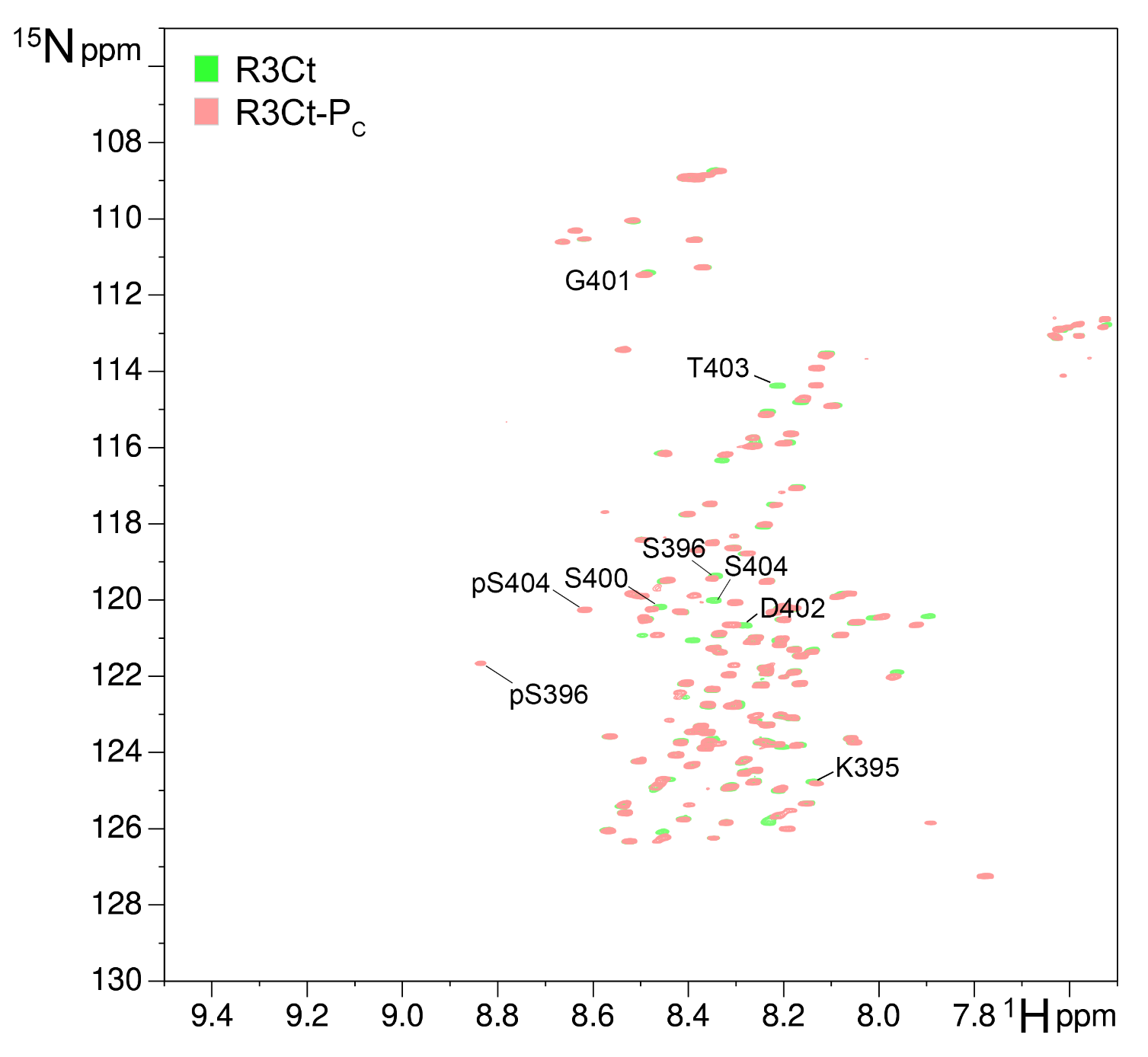


D


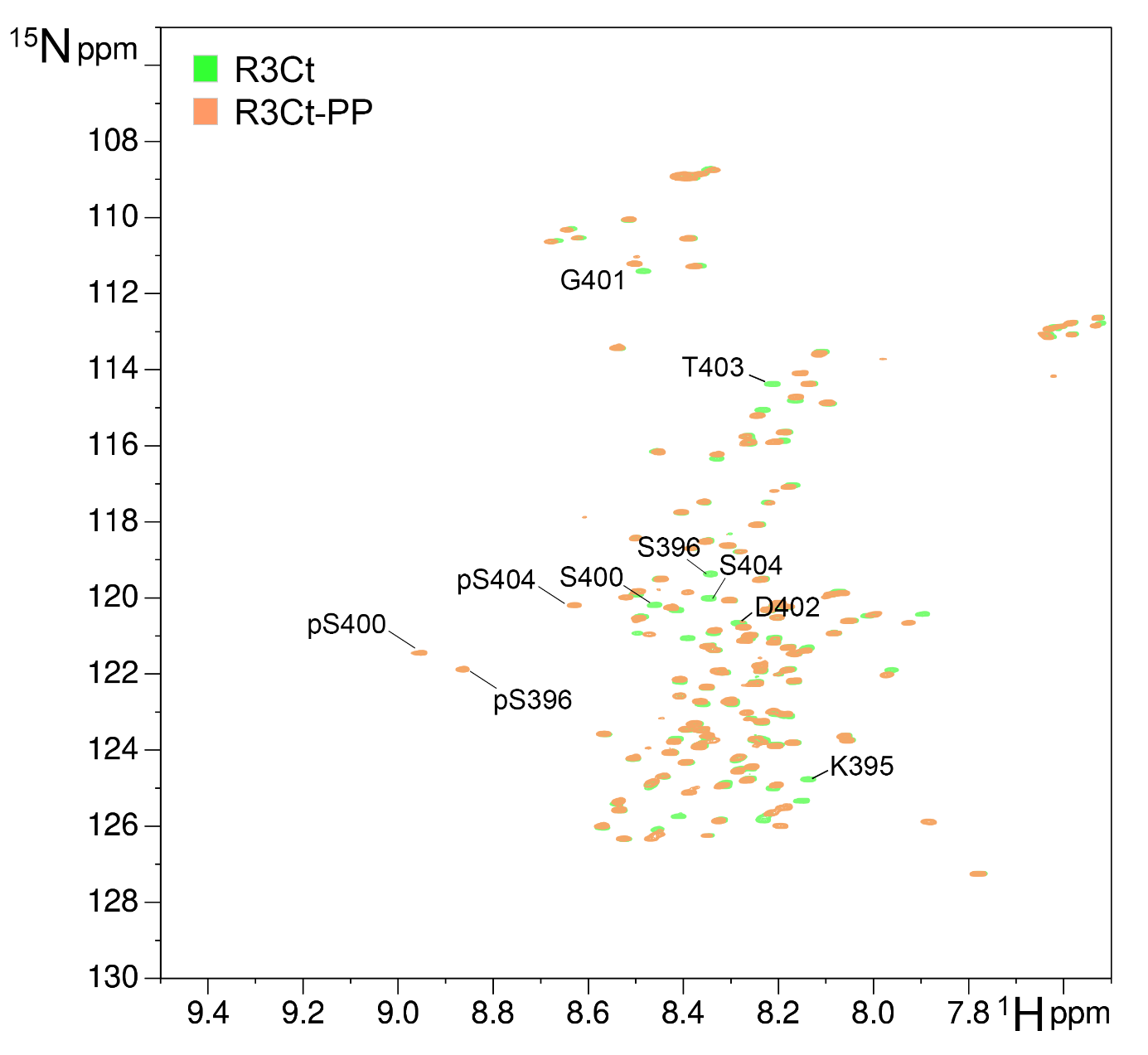


E


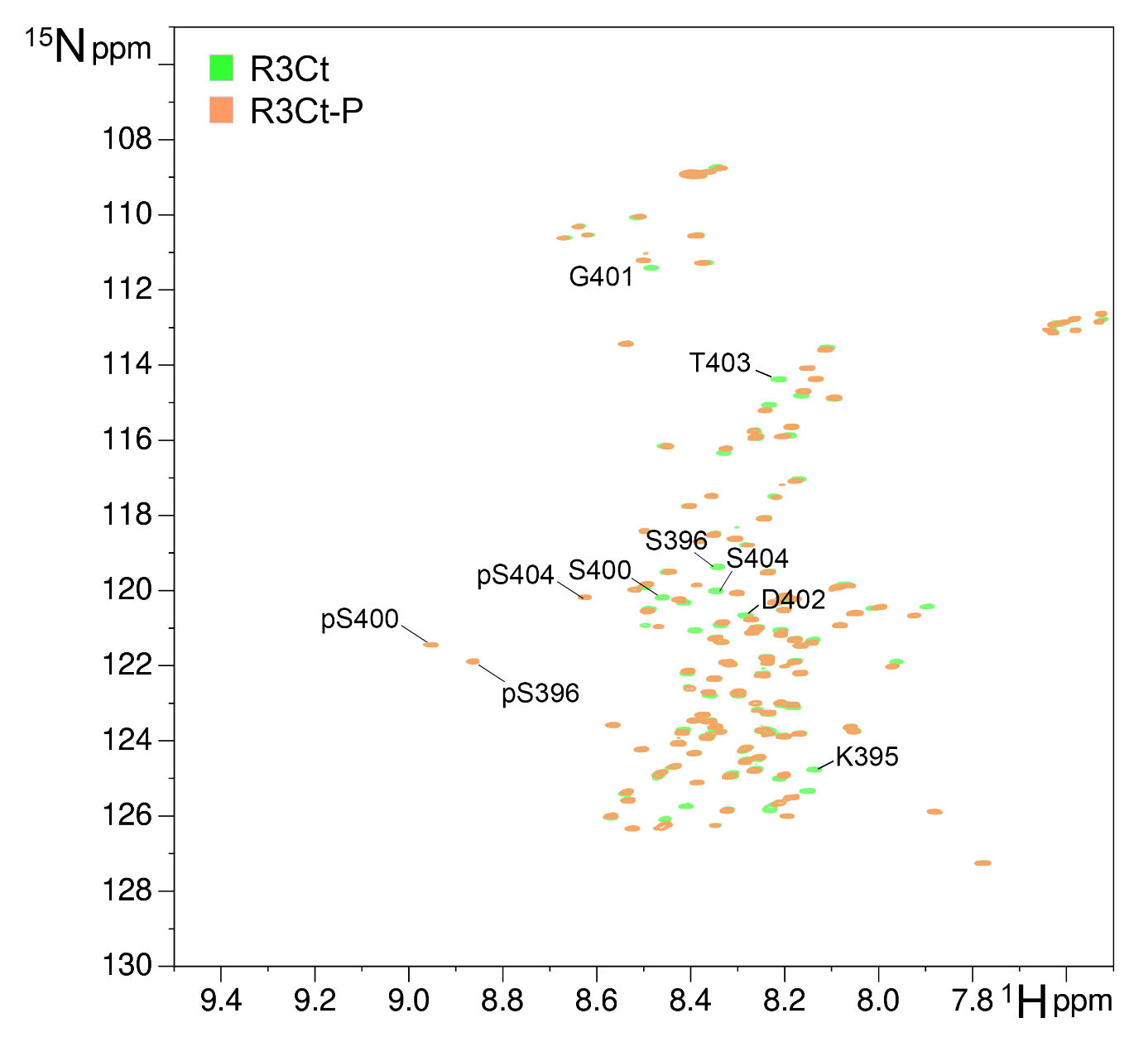


**Figure S1 :** Characterization of the phosphorylation patterns of the R3Ct fragment series used in this study. Mass spectrometry (MALDI-TOF) (A) and NMR (B-E) analyses of the R3Ct phosphorylation by CDK2 (P_C_) (light pink, A,B,C) or GSK3β (P) (orange, A,B,E), or the sequential phosphorylation by CDK2 and GSK3β (PP) (orange, A,B,D) are depicted with the control non-phosphorylated R3Ct (green, A-E).

**Figure S2**

A


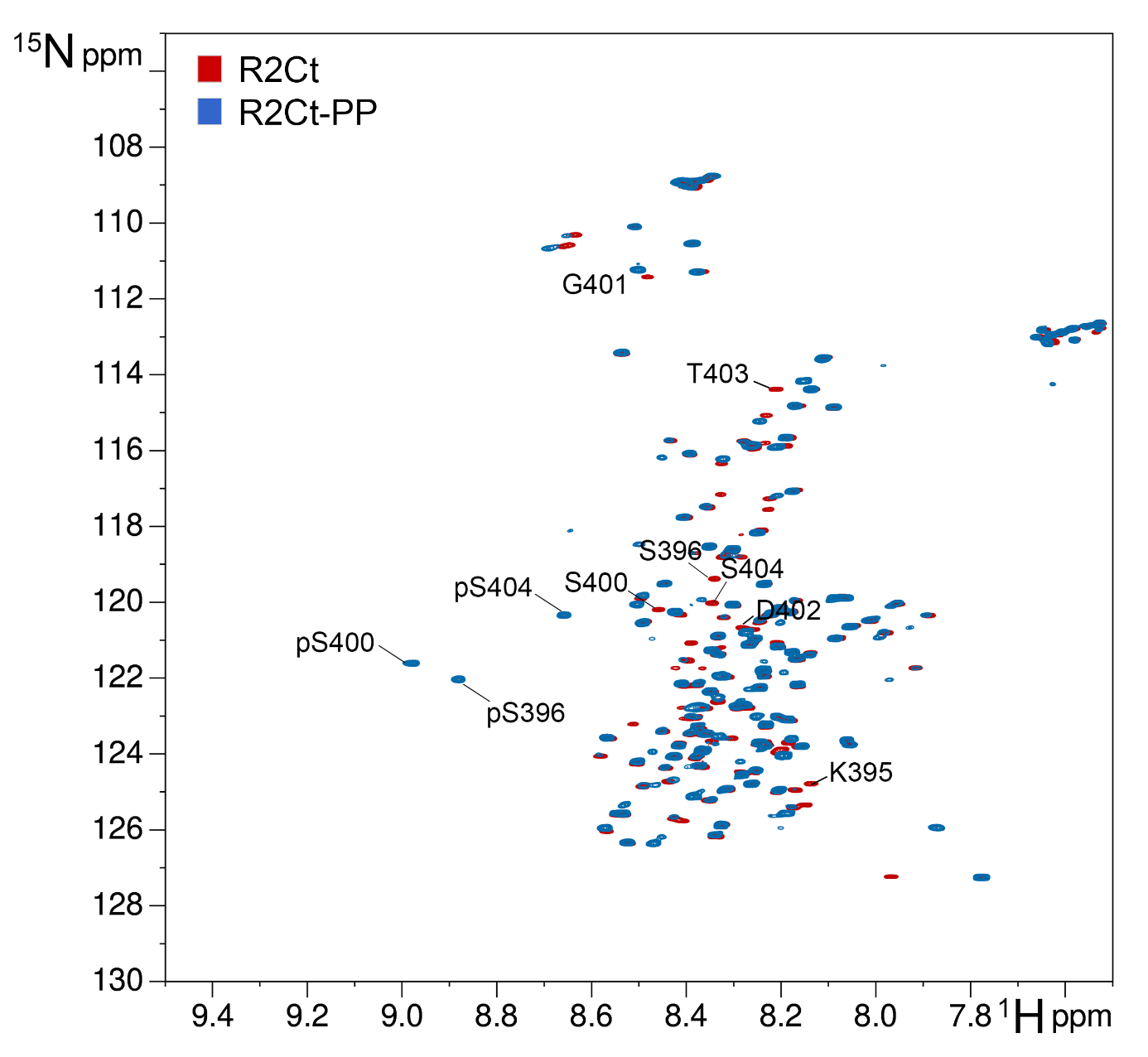


B


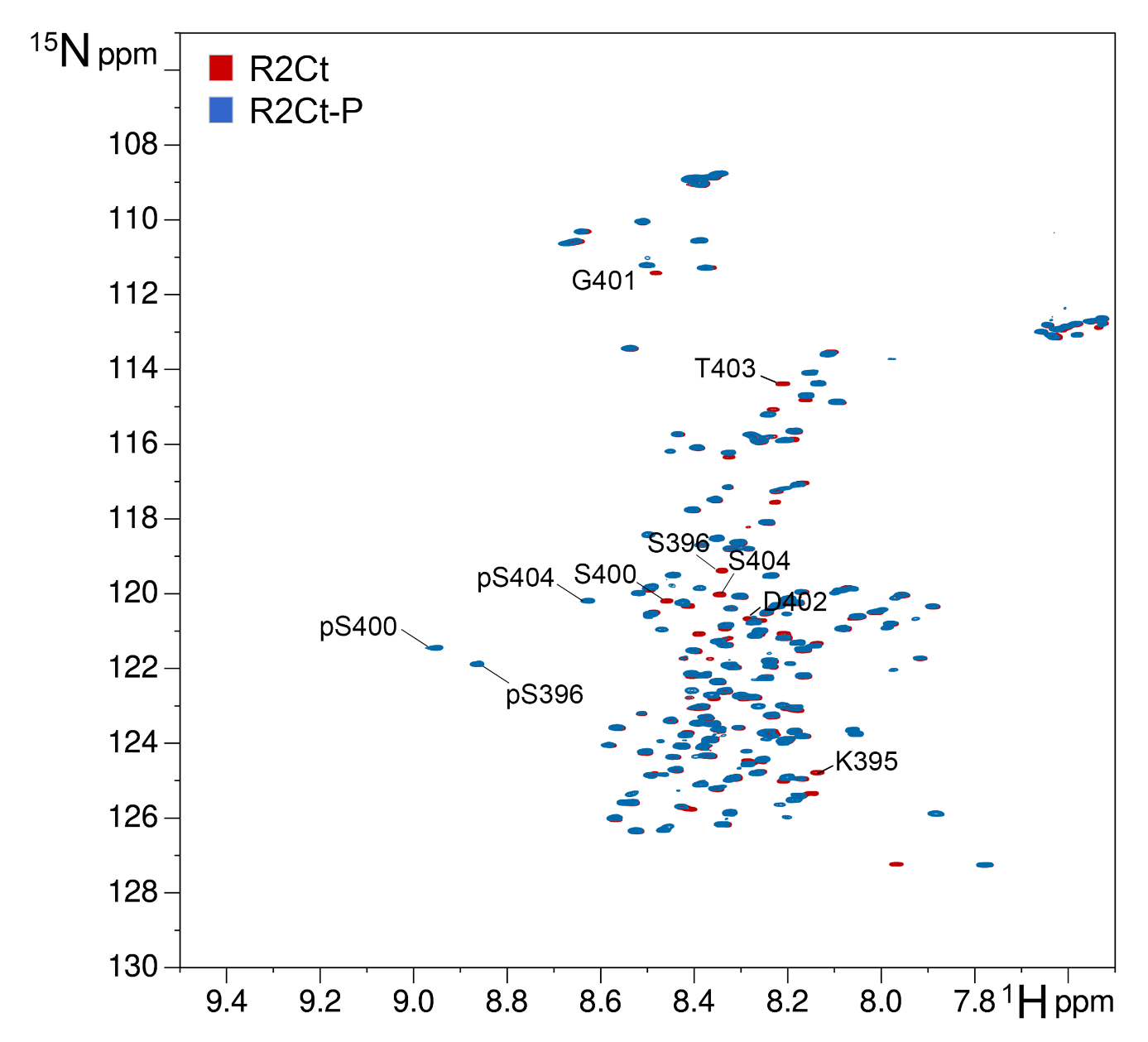


**Figure S2:** Characterization of the phosphorylation patterns of the R2Ct fragment series used in this study. NMR analyses of the R2Ct phosphorylation by GSK3β (P) (blue, B), or the sequential phosphorylation by CDK2 and GSK3β (PP) (blue, A) are depicted with the control non-phosphorylated R2Ct (red, A, B).

**Figure S3**


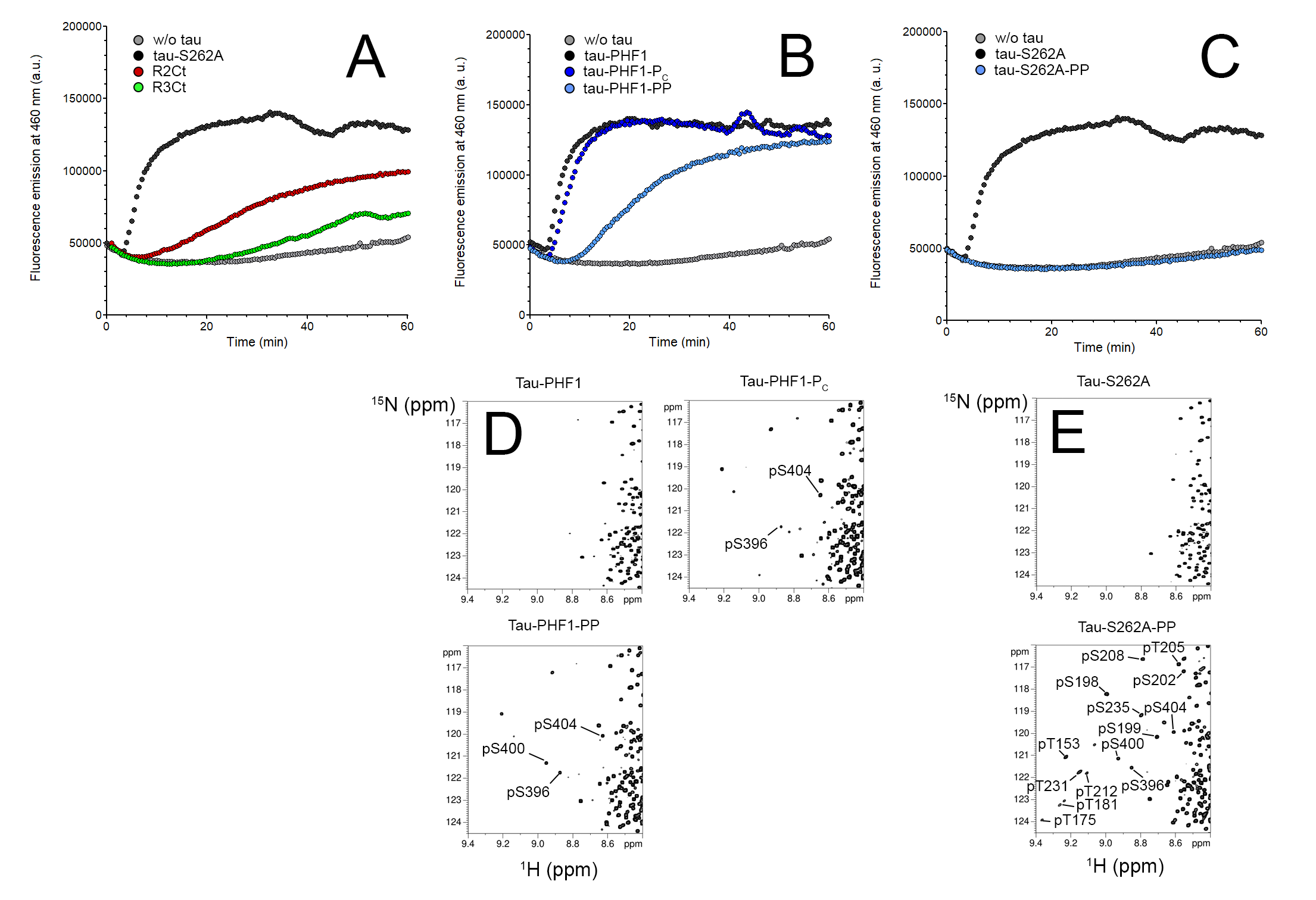


**Figure S3 :** Tubulin polymerization assays of R2Ct and R3Ct fragments compared with full-length tau protein mutants with diverse phosphorylation patterns. (A) Kinetics of tubulin polymerization of tau R2Ct (red) and R3Ct (green) fragments compared to full-length 2N4R tau protein (black) or without tau (grey). (B) Kinetics of tubulin polymerization of 2N4R Tau-PHF1 mutant in which all phosphorylatable proline-directed Ser/Thr have been mutated except those of the PHF-1 epitope, in a non-phosphorylated (black), CDK2/cyclinA phosphorylated (dark blue) or CDK2/cyclinA and GSK3β phosphorylated form (light blue). (C) Kinetics of tubulin polymerization of 2N4R Tau-S262A in a non-phosphorylated (black) or CDK2/cyclinA and GSK3β phosphorylated form (light blue). (D, E) NMR-based characterization of phosphorylation sites in Tau-PHF1 (D) upon phosphorylation by CDK2/cyclin A alone (P_C_) or in Tau-PHF1 (E) and Tau-S262A (E) upon sequential phosphorylation by CDK2/cyclinA and GSK3β (PP).

**Figure S4**


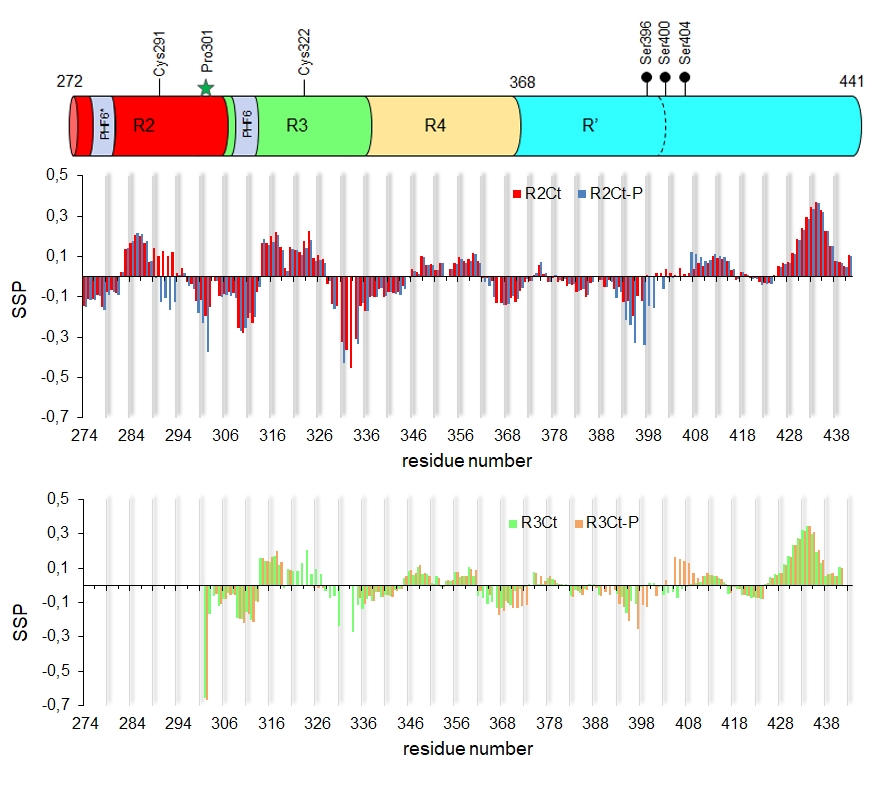


**Figure S4:** Secondary structure propensity calculated based on the Cα, Cβ and Hα chemical shifts for R2Ct in its non-phosphorylated (red) or GSK3β-phosphorylated (blue) forms, or R3Ct in its non-phosphorylated (green) or GSK3β-phosphorylated (orange) forms. The fragment domains are shown on the upper panel indicating the position of the two cysteine residues, the P301 site and the PHF-1 phosphorylation site along the sequence.

**Figure S5**


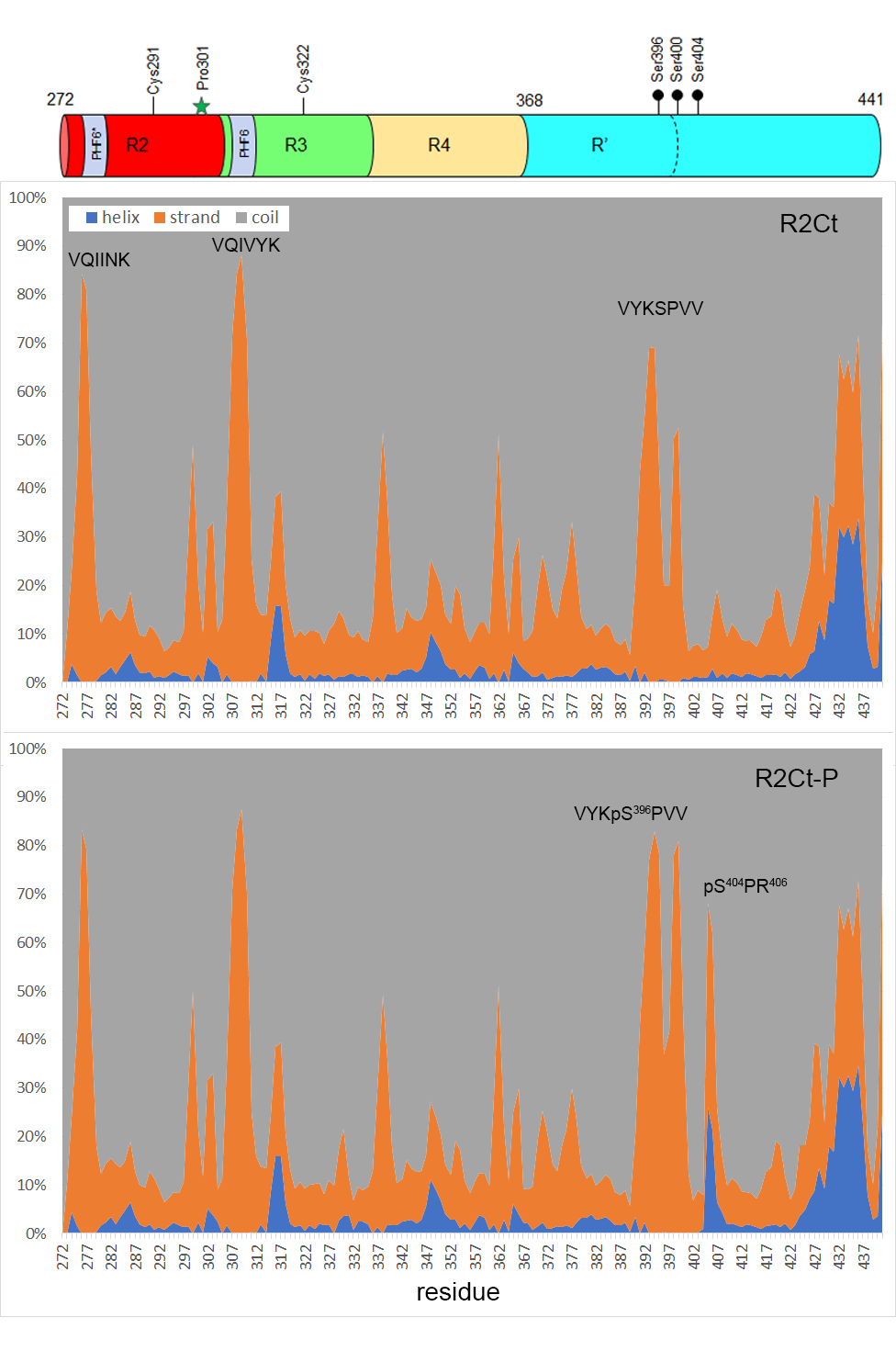


**Figure S5:** Protein backbone torsion angles predicted from NMR chemical shifts by Talos-N for the R2Ct and R2Ct-P fragments showing the cumulative fractions of helical (blue), extended strand (orange) and coil (grey) conformations. The chemical shift-based prediction indicates a higher content of β-strand in both R2 and R3 repeats related to R4 and R’ repeats, and a tendency of helical conformation in the extreme C-terminus (residues 427-437), independent of the phosphorylation state. A higher content of β-strand in the phosphorylated region of PHF-1 site (residues 390-400), comparable to the β-strand propensity of R2 and R3 repeats is observed. However, a transient helical/turn conformation is detected around the phospho-Ser404 residue. The fragment domains are shown on the upper panel indicating the position of the two cysteine residues, the P301 site and the PHF-1 phosphorylation site along the sequence.

**Figure S6**

A

**
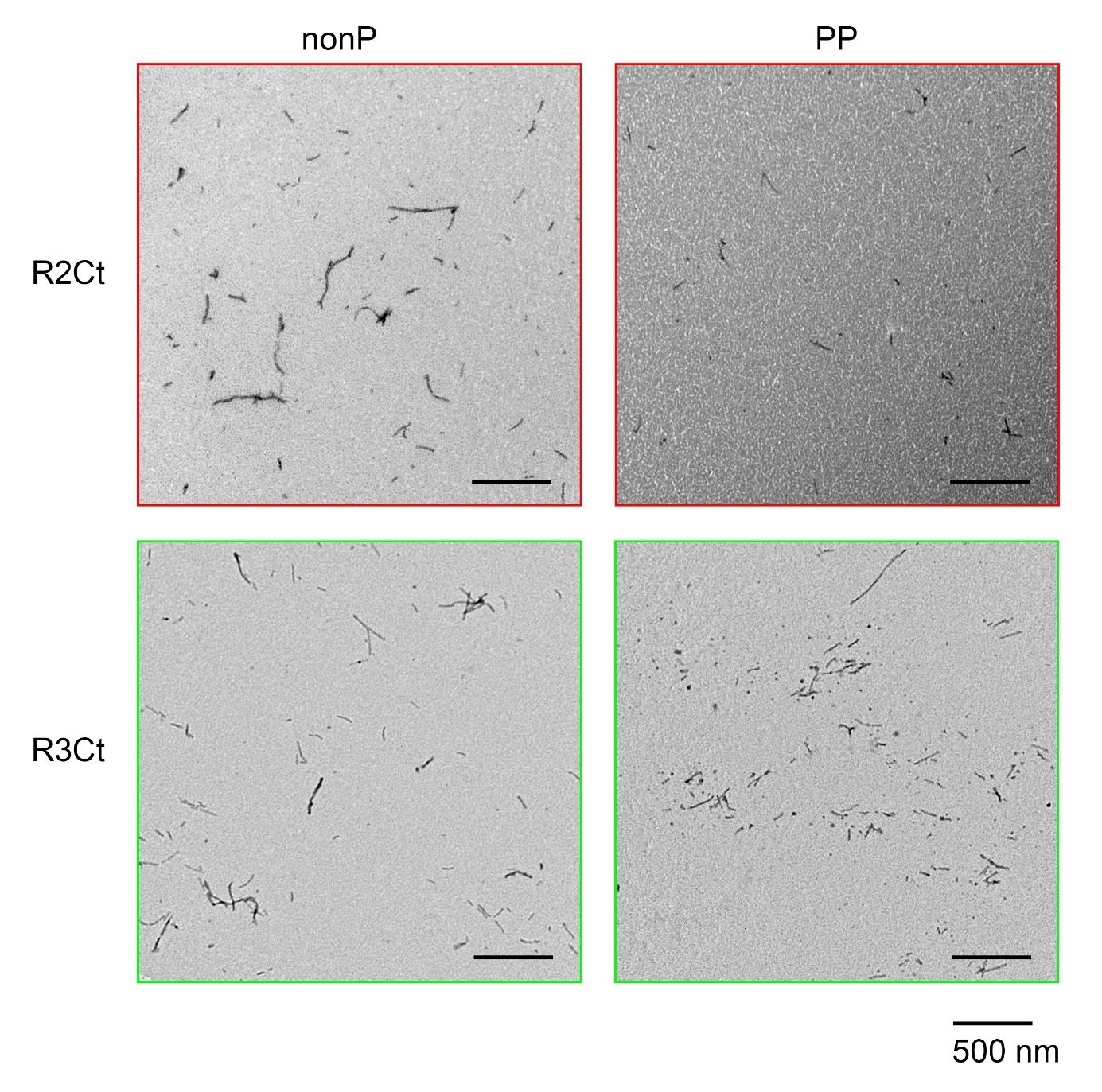
**

B

**
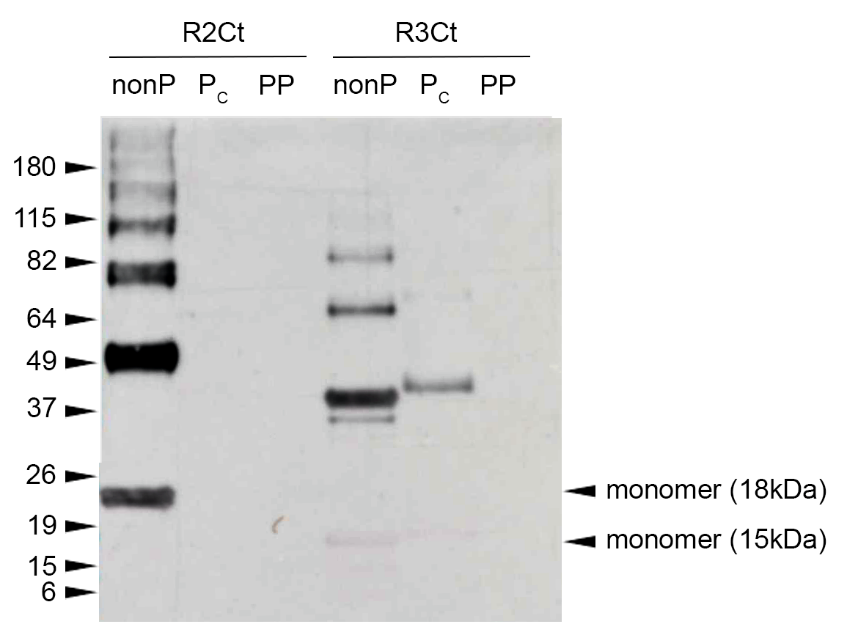
**

**Figure S6:** (A) TEM images of seeds from non-phosphorylated (nonP) or CDK2/GSK3β (PP) R2Ct (red) and R3Ct (green) fragments induced by heparin at a 10:1 tau:heparin ratio, which are further used in cross-seeding reactions. (B) Sedimentation assay of the aggregation reactions to produce R2Ct and R3Ct seeds, phosphorylated or not (immunodetected with the htau-3R antibody), showing the formation of dimers, trimers, etc… and high-molecular weight species in the soluble fraction of the aggregation mixtures. Note that the presence of a single cysteine, C322, in R3Ct indicates that it self-associates into multimers (higher than dimers) through non-covalent interactions between monomers or dimers. In contrast, the presence of two cysteines, C291 and C322, in R2Ct may eventually involve a cross-linking between cysteine residues of different monomers.

**Figure S7**


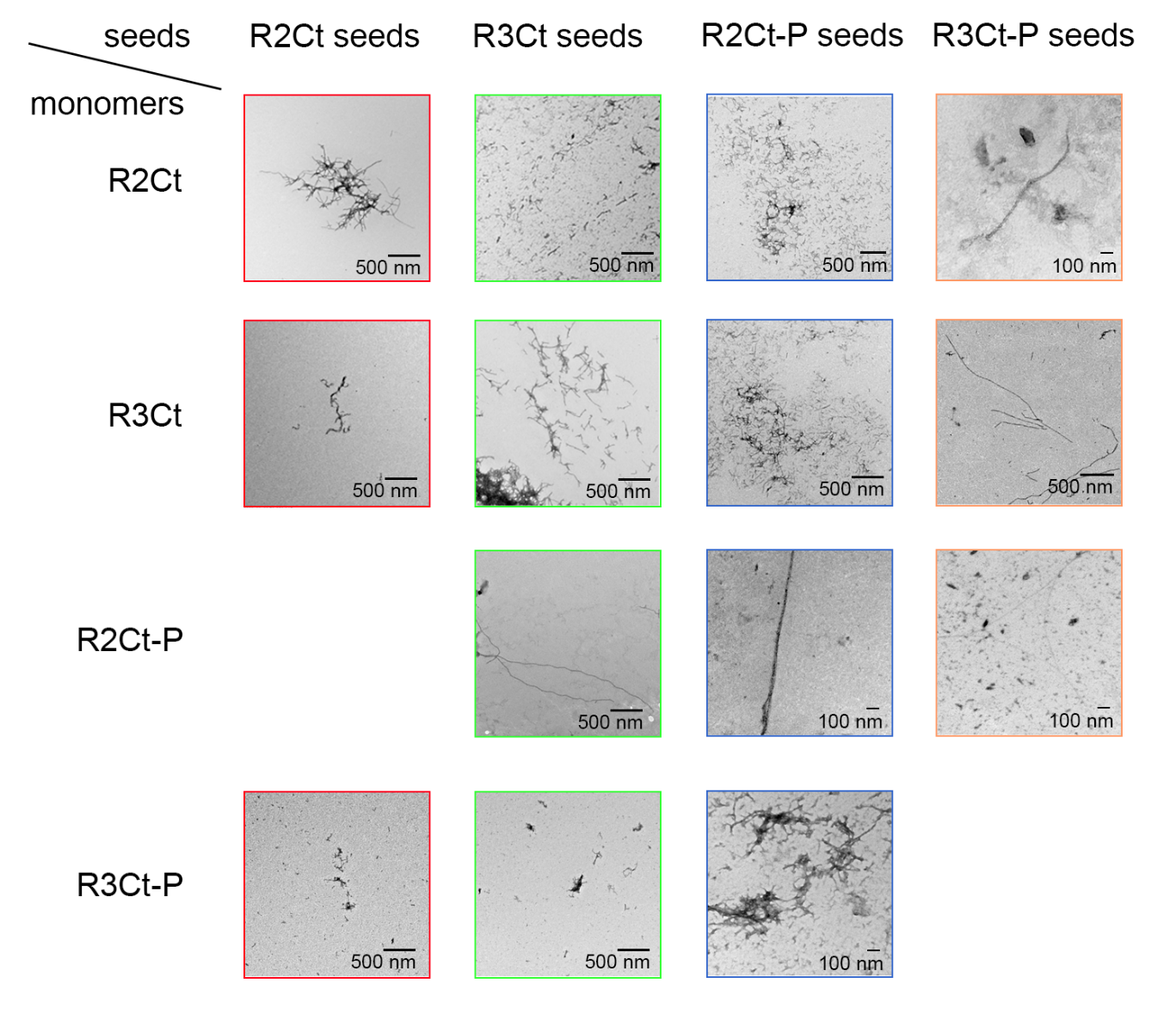


**Figure S7:** Negative staining images from TEM of every combination of seeding/cross-seeding reactions by non-phosphorylated (red) or phosphorylated (blue) seeds of R2Ct, or non-phosphorylated (green) or phosphorylated (orange) seeds of R3Ct on either R2Ct, R3Ct, R2Ct-P or R3Ct-P monomers.

**Figure S8**


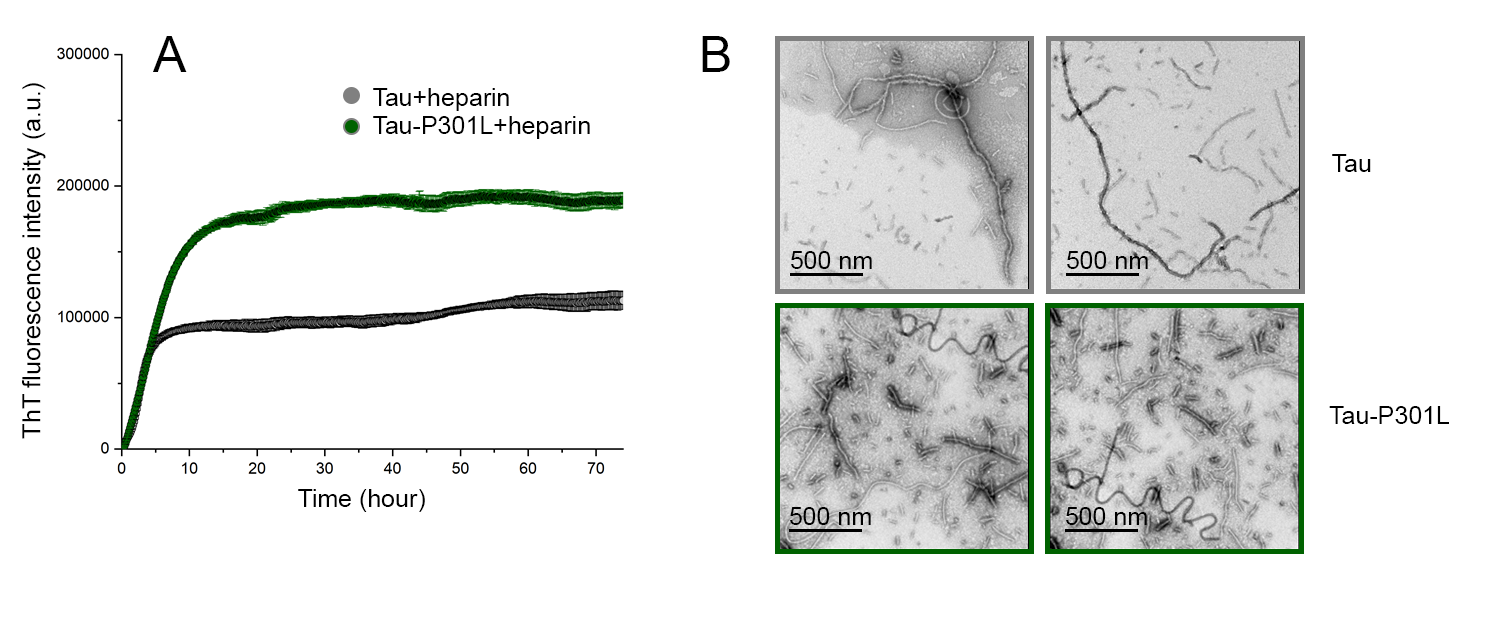


**Figure S8:** Aggregation of tau (grey) and tau-P301L mutant (green) induced by heparin shown by a time-dependent increase of ThT fluorescence (A) and transmission electron microscopy (B).

**Figure S9**


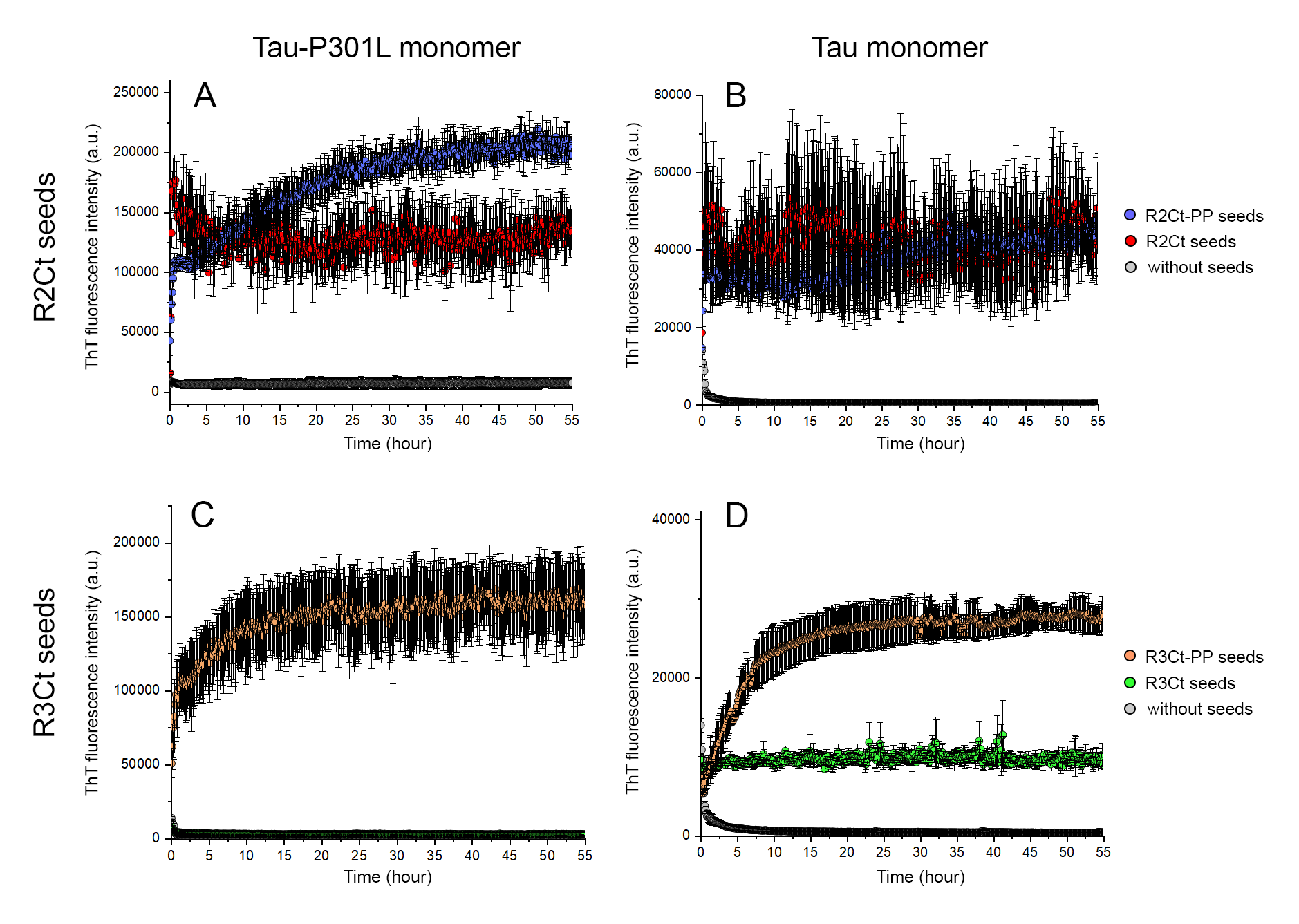


**Figure S9:** Kinetics of tau-P301L (A, C) and tau (B, D) aggregation induced by either R2Ct (red), R2Ct-P (blue), R3Ct (green) or R3Ct-P (orange) seeds followed by ThT fluorescence intensity depicted as means of two replicates ± SEM. Controls without seeds are indicated (grey).

**Figure S10**


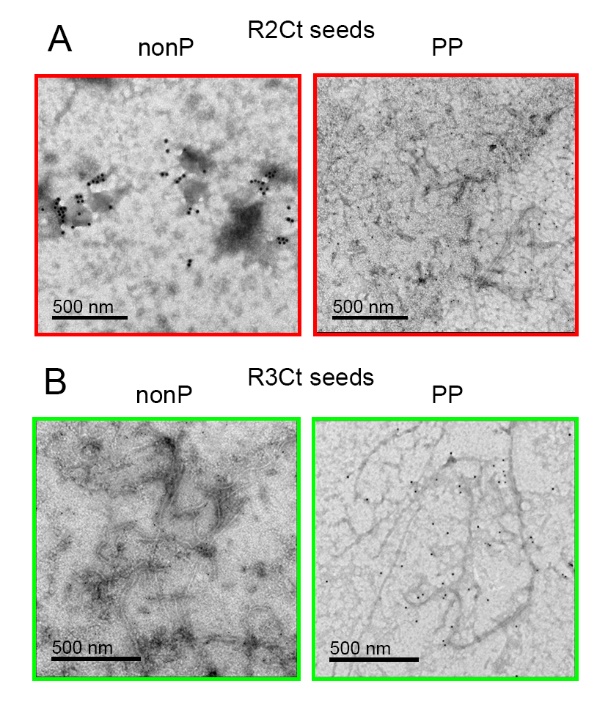


**Figure S10:** Immunogold labelling (with 6 nm-diameter gold particles) using HT-7 monoclonal antibody of tau-P301L seeding reactions with (A) R2Ct (red) or (B) R3Ct (green) seeds in either their non-phosphorylated (nonP) or CDK2/GSK3β-phosphorylated (PP) forms.

**Figure S11**


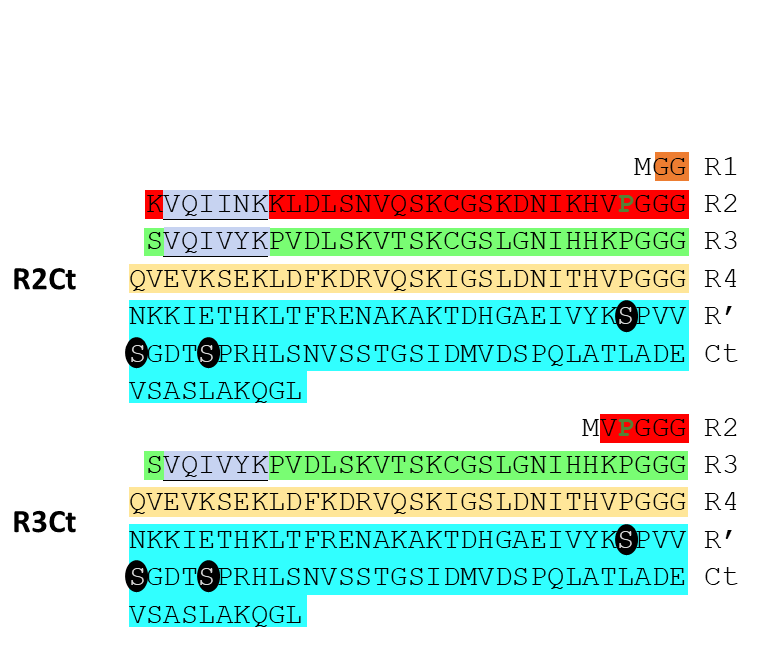


**Figure S11:** Protein sequences of R2Ct and R3Ct fragments used in this study, where phospho-serine amino acids of the PHF-1 phospho-epitope, phosphorylated by GSK3*β*, are indicated as black circles and the P301 site in green character. The PHF6* and PHF6 hexapeptide sequences are annotated in underlined characters and highlighted in grey. The color coding for the repeat, pseudo repeat or C-terminal sequences is the same as in Figure 1.

**Figure S12**

A

**
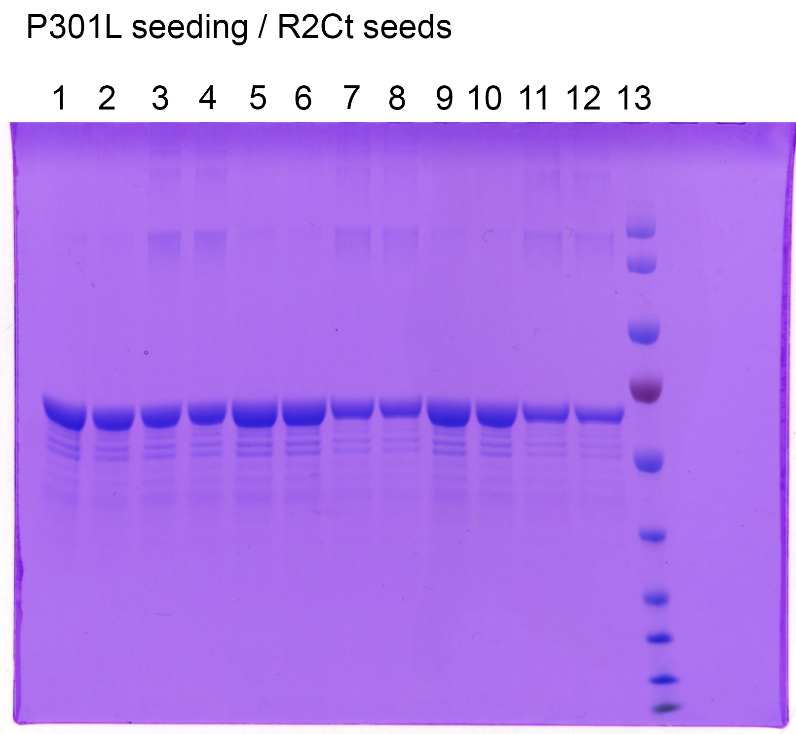
**

B

**
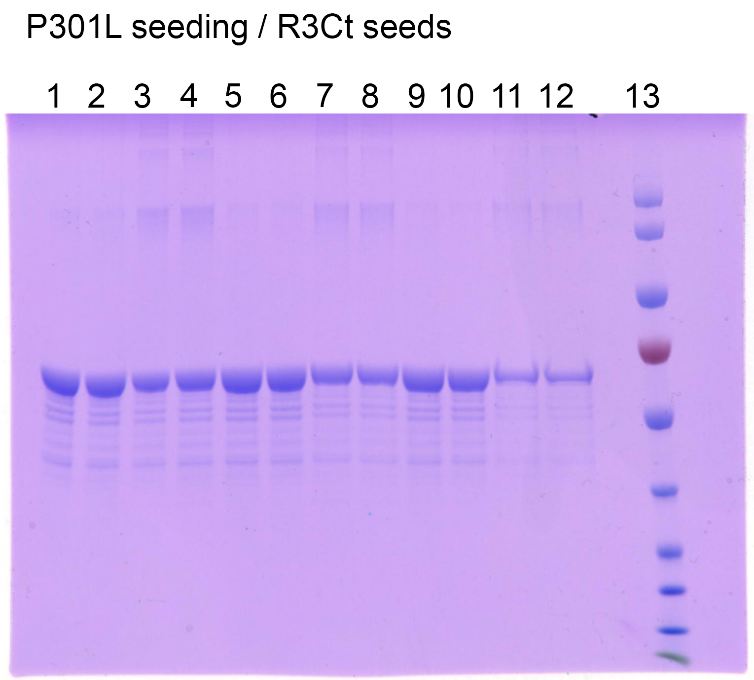
**

C

**
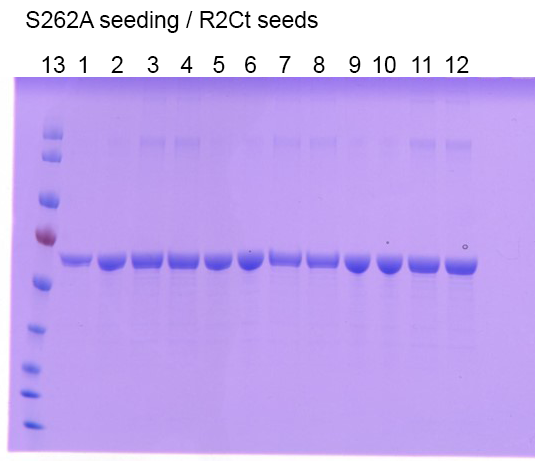
**

D

**
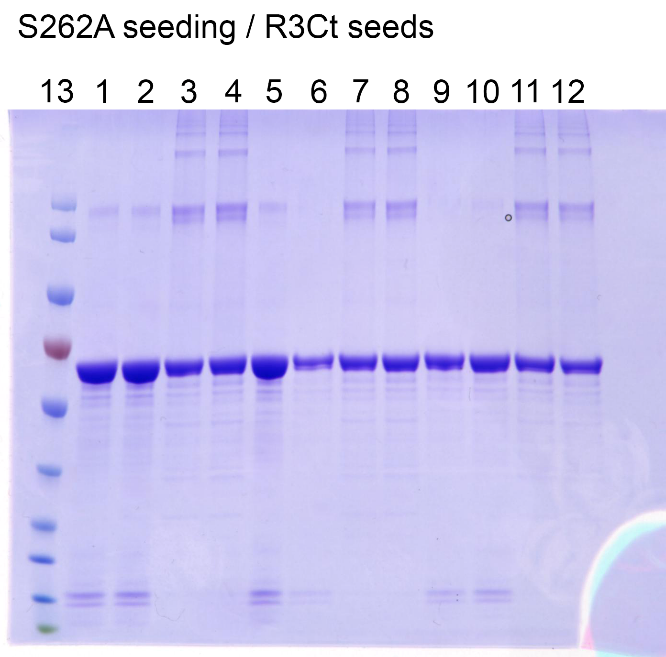
**

**Figure S12:** Full-length gels from Figure 5 showing the sedimentation assays of (A) seeding of tau P301L by R2Ct seeds, (B) seeding of tau P301L by R3Ct seeds, (C) seeding of tau S262A by R2Ct seeds, (D) seeding of tau S262A by R3Ct seeds. Samples of tau monomers (either tau P301L or S262A) at 25 μM incubated at 37°C with 1 μM of non-phosphorylated seeds are shown in duplicates at t_0_ (lanes 1,2) or t_200_ (lanes 3,4), with CDK2-phosphorylated seeds at t_0_ (lanes 5,6) or t_200_ (lanes 7,8), and with CDK2/GSK3β-phosphorylated seeds at t_0_ (lanes 9,10) or t_200_ (lanes 11,12). Protein molecular weight markers (ranging from 180 to 10 kDa) are indicated in lane 13.
